# Appetite loss in patients with motor neuron disease: impact on weight loss and neural correlates of visual food cues

**DOI:** 10.1093/braincomms/fcaf111

**Published:** 2025-03-14

**Authors:** Jeryn Chang, Thomas B Shaw, Pamela A McCombe, Robert D Henderson, Diana Lucia, Christine C Guo, Jinglei Lv, Kelly Garner, Saskia Bollmann, Shyuan T Ngo, Frederik J Steyn

**Affiliations:** School of Biomedical Sciences, Faculty of Medicine, The University of Queensland, Brisbane, QLD 4072, Australia; School of Electrical Engineering and Computer Science, The University of Queensland, Brisbane, QLD 4072, Australia; Centre for Advanced Imaging, The University of Queensland, Brisbane, QLD 4072, Australia; Department of Neurology, Royal Brisbane and Women’s Hospital, Brisbane, QLD 4006, Australia; Department of Neurology, Royal Brisbane and Women’s Hospital, Brisbane, QLD 4006, Australia; UQ Centre for Clinical Research, The University of Queensland, Brisbane, QLD 4006, Australia; Wesley Medical Research, The Wesley Hospital, Brisbane, QLD 4066, Australia; Department of Neurology, Royal Brisbane and Women’s Hospital, Brisbane, QLD 4006, Australia; UQ Centre for Clinical Research, The University of Queensland, Brisbane, QLD 4006, Australia; Wesley Medical Research, The Wesley Hospital, Brisbane, QLD 4066, Australia; Australian Institute of Bioengineering and Nanotechnology, The University of Queensland, Brisbane, QLD 4072, Australia; ActiGraph, LLC, Pensacola, FL 32502, USA; School of Biomedical Engineering, Faculty of Engineering, The University of Sydney, Sydney, NSW 2008, Australia; Brain and Mind Centre, The University of Sydney, Sydney, NSW 2050, Australia; School of Psychology, University of New South Wales, Sydney, NSW 2033, Australia; School of Electrical Engineering and Computer Science, The University of Queensland, Brisbane, QLD 4072, Australia; Department of Neurology, Royal Brisbane and Women’s Hospital, Brisbane, QLD 4006, Australia; UQ Centre for Clinical Research, The University of Queensland, Brisbane, QLD 4006, Australia; Wesley Medical Research, The Wesley Hospital, Brisbane, QLD 4066, Australia; Australian Institute of Bioengineering and Nanotechnology, The University of Queensland, Brisbane, QLD 4072, Australia; School of Biomedical Sciences, Faculty of Medicine, The University of Queensland, Brisbane, QLD 4072, Australia; Department of Neurology, Royal Brisbane and Women’s Hospital, Brisbane, QLD 4006, Australia; Wesley Medical Research, The Wesley Hospital, Brisbane, QLD 4066, Australia

**Keywords:** appetite, adiposity, fMRI, metabolism, MND

## Abstract

Motor Neuron Disease (MND) is associated with significant non-motor symptoms, including the loss of appetite. Loss of appetite has emerged as a dominant feature of the disease that may contribute to negative energy balance, faster disease progression and earlier death. We examined the prevalence and impact of appetite loss and analysed neural correlates of visual food stimuli with prandial status and appetite in people living with MND (plwMND). 157 plwMND and 120 non-neurodegenerative controls (NND Controls) were assessed for anthropometric, metabolic, appetite and clinical measures. Of these, 35 plwMND and 23 NND Controls underwent further functional MRI assessment of fasting and post-prandial responses to visual food cues. plwMND presented with reduced appetite (*P* < 0.001), with loss of appetite being more prevalent in plwMND than NND controls [OR = 2.59 (95% CI: = 1.46–4.61)]. Loss of appetite was not associated with hypermetabolism; however, was associated with fat mass loss (*P* < 0.05). Imaging assessment revealed no overall difference in response between plwMND and NND controls when viewing non-food and food images. In contrast, we found no prandial response in the temporal pole of plwMND compared with NND controls, and decreased activity in the cerebellum relative to appetite in plwMND. Loss of appetite, not hypermetabolism, contributes to negative energy balance in MND. Alterations in the temporal pole and cerebellum could contribute to altered appetite responses in some plwMND—brain regions not widely considered in appetite control—providing additional evidence to support widespread involvement of non-motor areas in the disease.

## Teaser

Chang *et al*. report that appetite loss is more prevalent in patients with Motor Neuron Disease than in controls and contributes to weight loss due to fat mass loss. Functional MRI reveals altered activity in the temporal pole and cerebellum, implicating brain regions not conventionally involved in non-motor aspects of the disease.

## Graphical Abstract

**Graphical Abstract:**
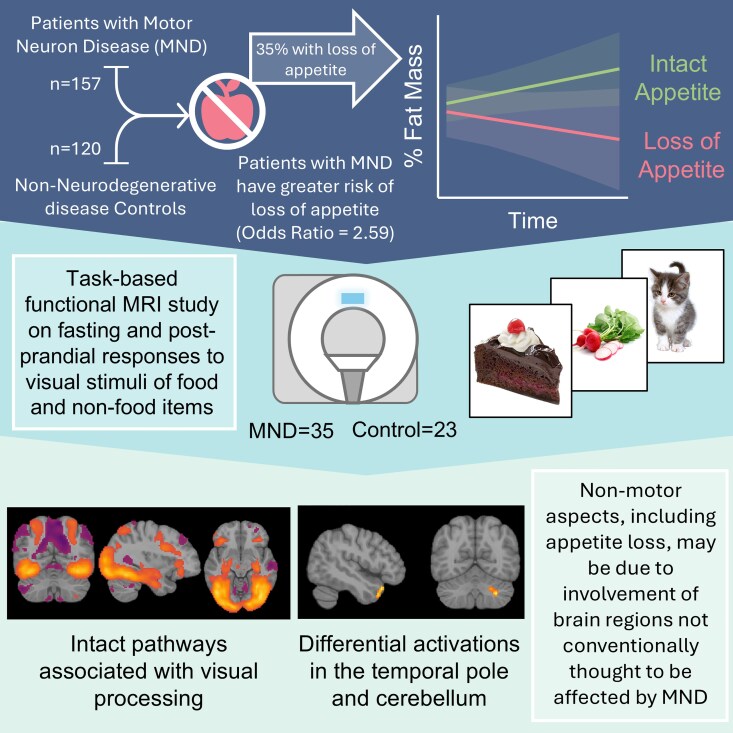

## Introduction

Motor Neuron Disease (MND) is a group of neurodegenerative diseases associated with the death of motor neurons in the motor cortex, brain stem, and/or spinal cord.^1^ While patients specifically present with motor deficits, it is recognized that significant non-motor symptoms, suggestive of a broader involvement of the central nervous system, contribute to the severity of the disease.^2^ The resulting non-motor manifestations are of clinical concern as they impact quality of life and disease progression. Studies have highlighted loss of appetite as an important non-motor symptom of the disease.^3,4^ This is associated with loss of body weight and fat mass^5^ which in turn contributes to faster disease progression and earlier death.^6^ Improved understanding of the factors that contribute to loss of appetite in MND can provide greater insight into the non-motor features of this disease and enhance the capacity to improve care, thereby improving the quality and duration of life for patients with MND.

Current evidence suggests that the loss of appetite in patients with MND may occur due to impairments in central mechanisms of appetite control, and more specifically, dysfunction of the hypothalamus,^7-10^ a critical integrator of metabolic and appetitive signalling. Metabolic and/or appetite disturbances in other neurodegenerative diseases are, however, thought to involve other brain regions^11,12^—a notion that has remained mostly unexplored in MND. That more extensive networks are involved is not unexpected. Reward systems associated with appetite control are highly interconnected with brain areas that contribute to the perception, evaluation, and pursuit of eating.^12,13^ For example, loss of appetite leading to lower energy intake, weight loss and worsening disease in Alzheimer's disease^14^ is associated with altered perfusivity in the orbitofrontal, anterior cingulate, and mesial temporal cortices.^15^ In patients with behavioural-variant frontotemporal dementia (bvFTD), increased caloric intake is associated with decreases in grey matter volume in the cingulate cortex, thalami and cerebellum,^11^ while increased preference for high-fat foods is associated with reductions in grey matter volume in the cingulate cortex, insula, putamen, amygdala and frontopolar regions.^16^ When considering amyotrophic lateral sclerosis (ALS; the most prevalent subtype of MND), TDP-43 inclusions are observed within the mesolimbic system including the striatum^17^ and fornix.^8^ Additionally, the amygdala volume is reduced.^18^ Collectively, these studies suggest that more extensive deficits in the neurocircuitry involved in the regulation of appetite control may occur in some patients with MND. It remains unclear, however, whether these deficits directly contribute to impairments in appetite and/or body weight regulation.

Here we examined the prevalence and impact of loss of appetite on body weight regulation in patients with MND and extended observations to consider the dysfunction of central pathways that could contribute to altered appetite control. Specifically, we assessed responses to visual stimuli of non-food and food items in the fasting and post-prandial state, and correlated outcomes from patients with MND to non-MND controls, and measures of appetite.

## Materials and methods

### Participants and study design

**Figure 1:**
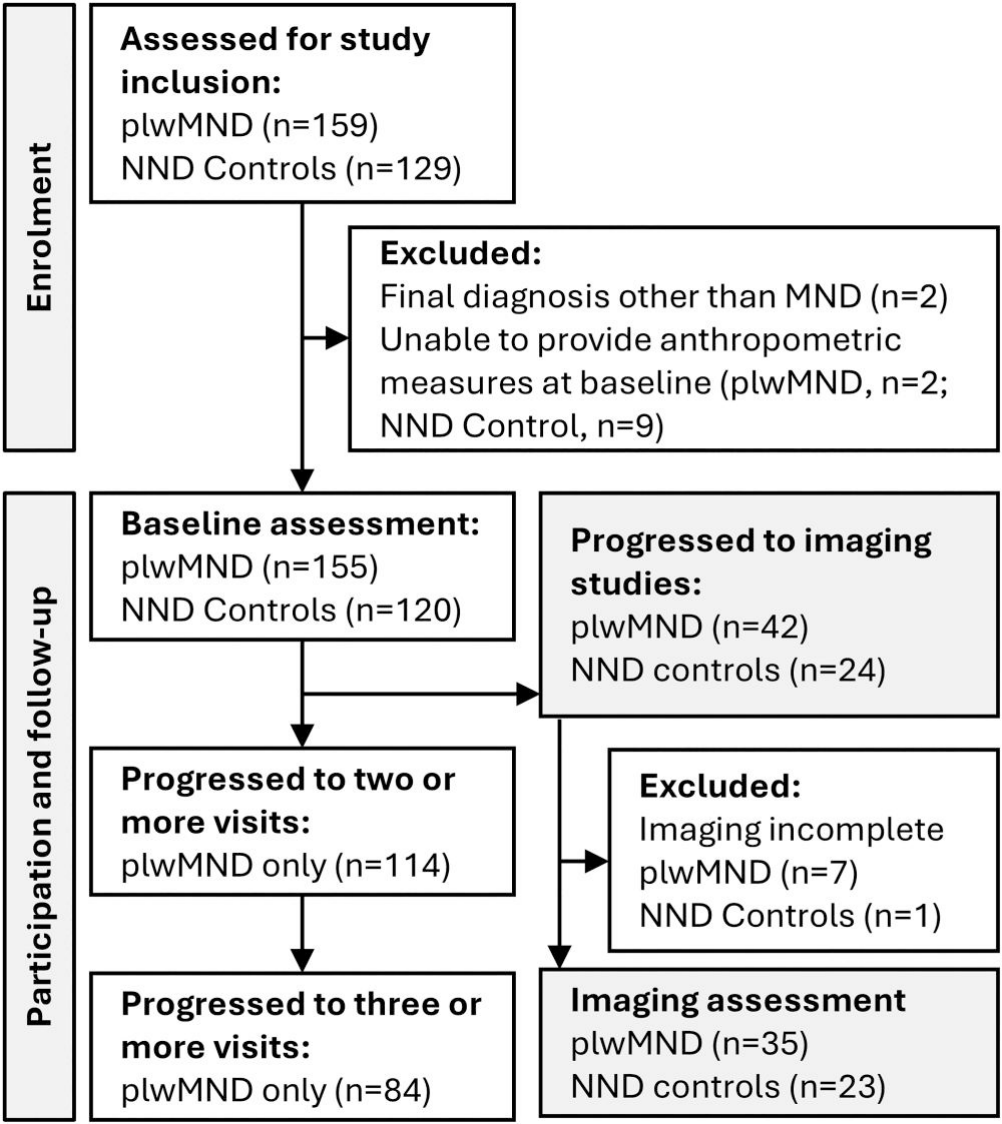
**Participant inclusion and follow-up.** Inclusion, baseline and follow-up participation of plwMND and NND controls.

One-hundred and fifty-nine patients with MND, approached via the MND research clinics at the Wesley Hospital and the Royal Brisbane and Women's Hospital (RBWH), participated in this cross-sectional and longitudinal case-control study (see Fig. 1). Inclusion criteria were patients with a diagnosis of MND aged between 18 and 80 years, with no history of gastrostomy and/or diabetes at the time of inclusion. Of these, 155 participants met the diagnostic criteria for possible, probable, or definite ALS,^19^ with three participants receiving an eventual diagnosis of ALS-bvFTD. Ten participants received an eventual diagnosis of PLS. Two participants received a final diagnosis other than MND and were excluded from further analysis. One-hundred and twenty-nine non-neurodegenerative disease control participants (NND Controls) were recruited as a convenience sample of spouses, friends or colleagues of people living with MND (plwMND). NND Controls had no history of cognitive or neurodegenerative disease. Longitudinal assessments interrogated changes in appetite, anthropometric, and clinical measures over time. One-hundred and fourteen plwMND completed two or more assessments. Assessments were repeated at 4.46 (2.97 SD) month intervals. NND controls provided baseline measurements only.

Sixty-two plwMND were invited to contribute to additional imaging studies. Of these, 42 progressed to assessment; 39 participants with a final diagnosis of ALS, two with a final diagnosis of PLS, and one with a final diagnosis of ALS-bvFTD. Four patients with ALS were noted to have some cognitive involvement following study inclusion. Seven participants were unable to complete all imaging assessments. Imaging studies were conducted at the Herston Imaging Research Facility at the RBWH. Twenty-four NND control participants contributed to imaging studies. Of these, one NND control participant was unable to complete all imaging assessments. Patients with respiratory dysfunction are at risk of not safely completing MRI studies, and so participants with a forced vital capacity <60% of predicted were not approached for imaging studies.

This study was approved by the University of Queensland, the RBWH (HREC/17/QRBW/616), and Uniting Care Health Human Research Ethics (Ref no. 1801) Committees. The privacy rights of all participants were observed, and all participants provided written and informed consent.

### Metabolic, anthropometric and clinical assessments

Anthropometric and metabolic measures were conducted and calculated as previously described.^20^ Five plwMND and five NND Controls had no baseline metabolic data available. Participants were asked to fast overnight (12-h) prior to assessment. Starting at 8 a.m., body composition was determined using air displacement plethysmography using the Cosmed BodPod Gold Standard system^21^; fat mass and fat-free mass were derived using the Siri algorithm.^22^ Measured resting energy expenditure (REE) was determined by indirect calorimetry (Quark RM respirometer, Cosmed),^21^ and values of fat-free mass were used to predict REE.^20^ Participants completed the Council of Nutrition Appetite Questionnaire (CNAQ)^23^; a CNAQ score of <29 was used to identify participants with loss of appetite, and has been used to demonstrate loss of appetite corresponding with weight^24^ and fat mass loss^5^ in MND. For MND patients, clinical history, including time since symptom onset, site of onset, diagnostic delay, and riluzole use was noted. The ALSFRS-R^25^ was implemented as a measure for functional capacity and was used to determine the change in ALSFRS-R since onset (ΔFRS).

### Imaging study protocol

**Figure 2:**
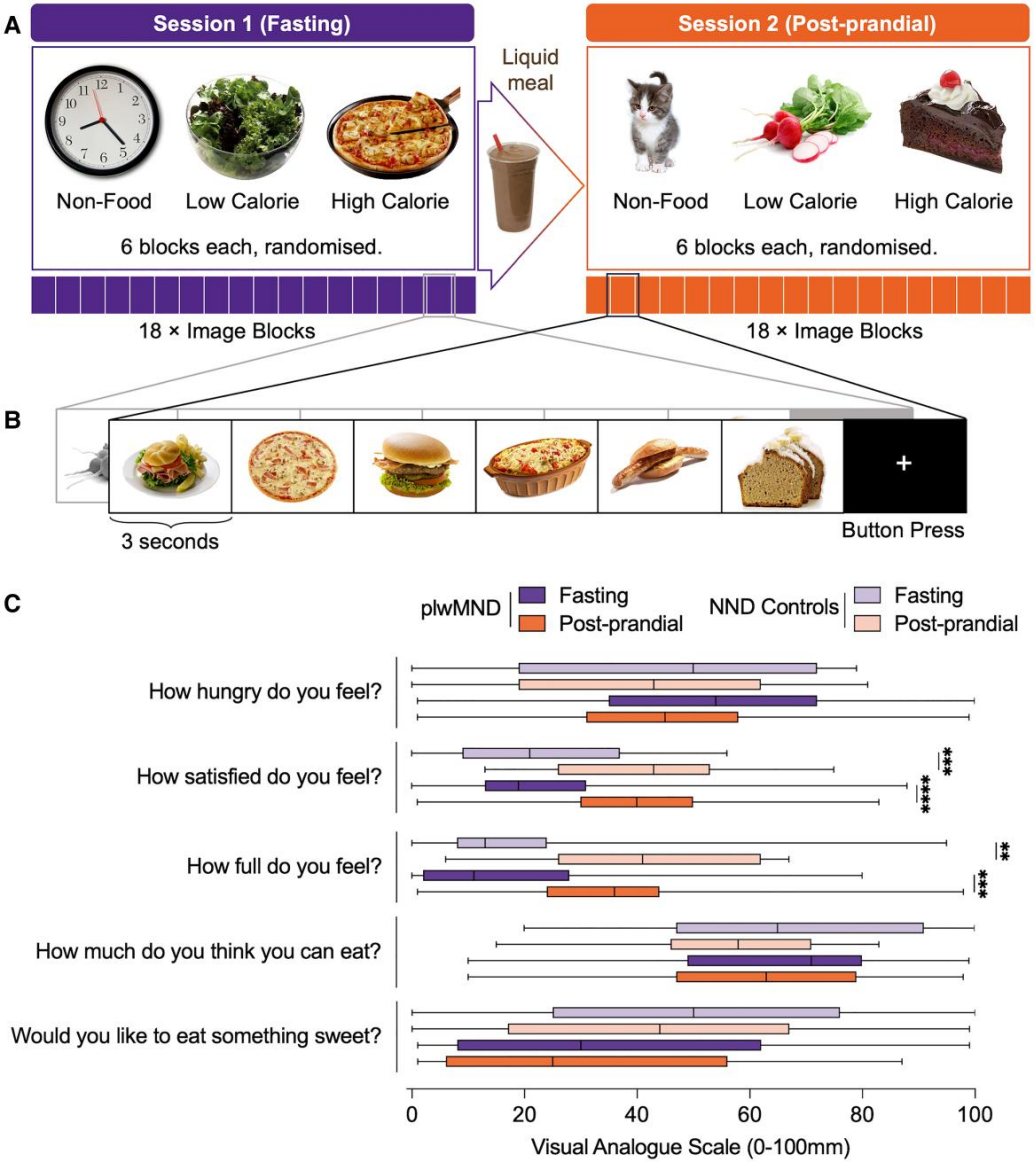
**Experimental setup and meal effect.** (**A**) Participants underwent two imaging sessions, one under fasting conditions, and the second after the consumption of a liquid mixed meal. Each session consisted of 18 image blocks, with each block showing a randomized category of either non-food, low-calorie food or high-calorie food items. (**B**) Each block comprises six images, shown for 3 s each. After each block, a fixation cross was displayed, where participants were asked to press a button. (**C**) Subjective appetite of plwMND (plwMND; *N* = 35) and NND controls (*N* = 23) on the VAS under fasting and post-prandial state. Multiple group comparisons were conducted using the Kruskal–Wallis test followed by the Wilcoxon Rank Sum test for pairwise comparisons. **P < 0.01 ***P < 0.001 ****P < 0.0001.

Participants fasted (nil by mouth, other than water) for 12 h prior to attending imaging assessments and completed two consecutive imaging sessions, each ∼45 min in duration (Fig. 2A). At arrival (8 a.m.) and prior to their first MRI session, participants completed the CNAQ and part one of a Visual Analogue Scale (VAS) to assess appetite.^26^ Participants were positioned in the MRI machine and instructed to observe visual stimuli during scanning. After the first MRI assessment, participants consumed a liquid meal (Sustagen®, Nestle; 15 kJ/kg.Bw; Protein—0.18 g/kg.Bw, Fat—0.05 g/kg.Bw, Carbohydrates—0.61 g/kg.Bw), before again completing the VAS for repeat assessment of subjective measures of appetite and palatability of the test meal.^26^ The elapsed time between imaging sessions was 33 ± 8 min, and the elapsed time between the consumption of the liquid meal and the start of the second imaging session was 29 ± 12 min (see Supplementary methods for full scanning parameters).

### Imaging experimental design

The imaging task included the presentation of 3 image categories on a projector screen mirrored to the participants’ field of view: high-calorie food, low-calorie food and non-food items (Fig. 2A). All images were obtained from the FoodPics Database.^27^ Image sets used for fasting and post-prandial response testing (Supplementary Table 1) were matched for complexity, size, brightness, calorie content and macronutrient balance (Supplementary Table 2). During each functional scan, 18 blocks of images, consisting of six blocks of each category, were presented in a random order. Each block included six randomly chosen images from each category, with each image displayed for 3 s. Following each block, a fixation cross was shown on the screen where participants were instructed to respond by pressing a button (Fig. 2B). The fixation cross was displayed for 14–17 s between blocks as a baseline. Button press responses were recorded but not included for analysis; responses confirmed that participants were alert and observing visual stimuli.

### fMRI contrasts

Full details of the fMRI processing and analysis can be found in the Supplementary methods.^28^ In our first-level analysis, we sought to estimate the following image contrasts: Non-food items, food > non-food items, high-calorie food > non-food. These contrasts were analysed with fasting and post-prandial states combined, under a fasting state, and between the fasting and post-prandial state. In addition, to understand how baseline appetite is associated with blood oxygen level-dependent (BOLD) response, we added CNAQ as a covariate of interest. For our second-level analysis, we analysed the main effects of the first-level contrasts in controls and plwMND. In addition, we assessed the contrast, Control > plwMND.

Significant cluster peaks were labelled based on the Automated Anatomical Labelling atlas.^29^ To identify significant clusters, we used a voxel-wise *P*-value threshold of 0.001, uncorrected. Clusters were considered significant if their family-wise error rate corrected *P*-values were below 0.05; only corrected values are presented throughout this manuscript. fMRI visualisations presented in this paper were generated using Nilearn.^30^

### Statistical analysis

Demography, anthropometry, and appetite were compared using the two-tailed Student *t*-test with Welch's correction for continuous variables. Chi-squared tests are conducted for categorical variables if all expected numbers are five or greater, otherwise, a Fisher's exact test is used. Longitudinal measures of body composition were modelled using a linear mixed effects model with participants and months treated as random effects. The alpha level was set at *P* < 0.05. We sought to ascertain group differences in measures of appetite, pre- and post-prandial, and to determine that MND-Control brain imaging contrasts could not be driven by demographic differences. Due to the non-normal distribution of data for perceptions of appetite at fasting and post-prandial, we used a Kruskal–Wallis test followed by a Wilcoxon Rank Sum test for subsequent pairwise comparisons for post-hoc testing.

## Results

### Participant demographics, anthropometry, measures of metabolism, appetite and clinical characteristics

A total of 275 participants, including 155 plwMND and 120 NND controls, provided baseline data. Demographic and clinical characteristics of patients and controls are summarized in Table 1. Cohorts were matched by weight (*P* = 0.54), BMI (*P* = 0.17), and fat mass (*P* = 0.54). Patients were older (*P* < 0.05) and were more likely to be male (*P* < 0.01). REE did not differ between cases or controls (*P* = 0.12); however, patients had an overall higher metabolic index (*P* < 0.01) and were more likely to be hypermetabolic (*P* < 0.01). Appetite scores, as defined by the CNAQ, were significantly lower in plwMND (29.12 versus 30.7, *P* < 0.01), with 35.5% of patients and 17.5% of NND controls having CNAQ scores indicative of loss of appetite (CNAQ < 29 pts). The crude OR for loss of appetite in plwMND was 2.59 (95% confidence interval: 1.46–4.61). When considering CNAQ sub-scores, patients reported overall lower responses for their general perception of appetite (*P* < 0.01), taste (in general and relative to childhood; *P* < 0.01), feeling sick or nauseated (*P* < 0.05), and mood (*P* < 0.05).

**Table 1:** Baseline characteristics of plwMND, and non-NND controls at time of baseline assessment

	Main cohort case-control comparison^a^			Imaging case-control comparison		
	MND (*n* = 155)	NND controls (*n* = 120)	*P* ^b^	MND (*n* = 35)	NND controls (*n* = 23)	*P* ^b^
**Demographics**						
Age (years)	60.03 (10.14)	56.89 (11.30)	<0.05	59.17 (8.95)	55.09 (11.04)	0.14
Sex (female)	41 (26.45)	56 (46.67)	<0.01	9 (25.71)	6 (26.09)	1.00
**Anthropometric measures**						
Weight (kg)	81.65 (18.07)	83.02 (18.54)	0.54	80.84 (16.06)	84.77 (20.36)	0.44
BMI (kg/m^2^)	27.03 (5.15)	27.98 (6.06)	0.17	27.15 (5.00)	27.32 (6.50)	0.92
Fat mass (kg)	28.44 (13.58)	29.42 (12.83)	0.54	28.36 (13.32)	26.59 (17.32)	0.68
Fat-free mass (kg)	53.2 (11.04)	53.14 (10.87)	0.96	52.60 (9.79)	58.20 (9.92)	<0.05
**Appetite and metabolic measures**						
CNAQ Total	29.12 (4.04)	30.7 (3.05)	<0.01	29.56 (3.10)	32 (3.06)	<0.01
Q1 (Appetite)	3.68 (1.08)	4.04 (0.87)	<0.01	3.67 (0.99)	4.35 (0.78)	<0.01
Q2 (Full)	3.7 (0.85)	3.84 (0.61)	0.13	3.58 (1.00)	4.04 (0.21)	<0.01
Q3 (Hunger)	2.68 (0.86)	2.72 (0.92)	0.75	2.92 (0.80)	3.09 (0.95)	0.48
Q4 (Taste)	4.1 (0.81)	4.33 (0.64)	<0.01	4.17 (0.77)	4.43 (0.73)	0.18
Q5 (Taste compared with childhood)	3.09 (0.71)	3.45 (0.77)	<0.01	3.14 (0.68)	3.43 (0.73)	0.13
Q6 (Normally I eat)	3.86 (0.63)	3.86 (0.66)	0.99	3.86 (0.64)	3.96 (0.77)	0.62
Q7 (Sick or Nauseated)	4.36 (0.88)	4.57 (0.62)	<0.05	4.64 (0.49)	4.74 (0.45)	0.42
Q8 (Mood)	3.66 (0.78)	3.89 (0.73)	<0.05	3.58 (0.73)	3.96 (0.64)	<0.05
REE (Kcal/day)^c^	1639.58 (365.03)	1569.77 (351.45)	0.12	1721.85 (372.62)	1731.26 (348.93)	0.92
Metabolic index^c^	1.01 (0.15)	0.97 (0.13)	<0.01	1.07 (0.2)	1.01 (0.11)	0.13
Hypermetabolic^c^	37 (24.67)	13 (11.30)	<0.01	13 (38.24)	5 (21.74)	0.31
**Clinical measures**						
ALSFRS-R total	37.25 (5.65)			37.06 (5.98)		
Bulbar	9.70 (2.43)			9.64 (2.13)		
Upper limb	6.16 (1.94)			5.91 (2.32)		
Lower limb	4.71 (2.12)			4.42 (2.22)		
Respiratory	10.67 (1.94)			11.52 (1.03)		
Diagnostic delay (months)	17.06 (18.89)			12.71 (7.42)		
Time since onset (months)	26.14 (26.77)			20.71 (13.63)		
ΔFRS	−0.59 (0.54)			−0.50 (0.35)		
Site of onset (Bulbar)	37 (23.87)			5 (14.29)		
**Final diagnosis**						
ALS/PLS/ALS-bvFTD	142/10/3			32/2/1		

ALS, amyotrophic lateral sclerosis; ALSFRS-R, ALS functional rating scale-revised; BMI, body mass index; bvFTD, behavioural-variant frontotemporal dementia; PBP, progressive bulbar palsy; PLS, primary lateral sclerosis; CNAQ, Council on Nutrition Appetite Questionnaire; ΔFRS, decline in the ALSFRS-R (points/month since date of symptom onset).

^a^Data presented as mean (SD) for continuous variables or *n* counts (%) for categorical variables.

^b^Student *t*-tests with Welch's correction is conducted for continuous variables. Chi-squared tests are conducted for categorical variables if all expected numbers are five or greater. If this condition is not met, a Fisher's exact test is used instead.

^c^Five plwMND and five NND Controls had no baseline metabolic data available.

Thirty-five plwMND and 23 NND controls completed imaging studies (Table 1). Cohorts were matched by age (*P* = 0.14), sex (*P* = 1.00), weight (*P* = 0.44), BMI (*P* = 0.92), and fat mass (*P* = 0.68). Fat-free mass was lower in plwMND (*P* < 0.05). CNAQ scores at baseline were significantly lower in plwMND (29.56 versus 32.00, *P* < 0.01), and 31.4% of patients who completed imaging studies presented with loss of appetite. Both groups indicated a significant increase in satisfaction and fullness following the consumption of the mixed liquid meal (Fig. 2C). There were no other differences in responses to the VAS between both groups, at fasting, and following the consumption of the mixed liquid meal.

### Comparison of patients with intact versus impaired appetite

At study inclusion, 55 patients reported loss of appetite (Table 2). Sex (*P* = 0.46), weight (*P* = 0.76), BMI (*P* = 0.79), fat mass (*P* = 0.96), and fat-free mass (*P* = 0.64) did not differ between plwMND reporting intact versus loss of appetite. While plwMND with loss of appetite were slightly older, this difference was not statistically significant (*P* = 0.06). Patients with loss of appetite scored lower on all CNAQ sub-scores, suggesting an overall decrease in all aspects of appetite. Under fasting conditions, patients who reported a loss of appetite scored higher on VAS questions on fullness (*P* < 0.05), and lower on questions on how much they feel they can eat (*P* < 0.01). In the post-prandial state, these participants continued to score lower (*P* < 0.01) on questions regarding how much they could eat; generally, patients reporting a loss of appetite reported they felt fuller and could eat less when fasting and post-prandial. Measured energy expenditure did not differ between groups (*P* = 0.50), and metabolic index and the prevalence of hypermetabolism were the same between groups. Patients who reported a loss of appetite had lower baseline ALSFRS-R scores (*P* < 0.05); lower limb (*P* = 0.05) and respiratory (*P* = 0.05) sub-scores. There was no clear association between cognition and appetite, with two of the four patients with some cognitive involvement also reporting a loss of appetite (*P* = 0.26).

**Table 2:** Within-case comparisons between plwMND with loss (CNAQ < 29) of appetite versus plwMND with intact (CNAQ ≥ 29) appetite

	Within-case comparison^a^		
	Loss of appetite CNAQ <29 (*n* = 55)	Intact appetite CNAQ ≥ 29 (*n* = 100)	*P* ^b^
**Demographics**			
Age (years)	61.95 (8.71)	58.97 (10.74)	0.06
Sex (female)	17 (31)	24 (24)	0.46
**Anthropometric measures**			
Weight (kg)	81.01 (20.86)	82 (16.44)	0.76
BMI (kg/m^2^)	27.20 (6.35)	26.94 (4.39)	0.79
Fat mass (kg)	28.35 (16.95)	28.49 (11.4)	0.96
Fat-free mass (kg)	52.65 (11.04)	53.51 (11.08)	0.64
**Appetite and metabolic measures**			
CNAQ total	24.98 (3.64)	31.39 (1.87)	<0.01
Q1 (Appetite)	2.67 (0.9)	4.23 (0.71)	<0.01
Q2 (Full)	3.15 (1.08)	4.01 (0.46)	<0.01
Q3 (Hunger)	2.07 (0.81)	3.01 (0.69)	<0.01
Q4 (Taste)	3.56 (0.79)	4.4 (0.65)	<0.01
Q5 (Taste compared with childhood)	2.82 (0.72)	3.24 (0.65)	<0.01
Q6 (Meal frequency)	3.69 (0.74)	3.95 (0.54)	<0.01
Q7 (Sick or Nauseated)	3.87 (1.06)	4.63 (0.63)	<0.05
Q8 (Mood)	3.18 (0.77)	3.92 (0.65)	<0.01
REE (kcal/day)^d^	1615.67 (348.76)	1657.62 (371.82)	0.50
Metabolic Index^d^	1.02 (0.13)	1.01 (0.16)	0.88
Hypermetabolic^d^	11 (21.57)	26 (26.26)	0.67
**Day of imaging appetite measures^c^**			
VAS (Fasting)			
Hungry	40.36 (27.05)	57.42 (22.39)	0.09
Satisfied	33.55 (24.71)	19.54 (16.72)	0.11
Full	34.27 (28.49)	13.83 (18.11)	<0.05
How much can you eat	52.55 (14.23)	73.21 (18.81)	<0.01
Sweet	63.45 (27.24)	63.71 (31.41)	0.98
VAS (post-prandial)			
Hungry	33.27 (20.63)	47.29 (20.78)	0.08
Satisfied	48.27 (17.68)	37.04 (16.29)	0.09
Full	45.81 (17.95)	33.25 (20.34)	0.08
How much can you eat	46.91 (16.57)	68.71 (17.82)	<0.01
Sweet	61.27 (22.95)	72.54 (27.06)	0.22
**Clinical measures**			
ALSFRS-R total	35.96 (5.13)	37.95 (5.81)	<0.05
Bulbar	9.22 (2.55)	9.96 (2.34)	0.08
Upper limb	6.33 (1.63)	6.07 (2.09)	0.39
Lower limb	4.23 (1.89)	4.99 (2.19)	<0.05
Respiratory	10.11 (2.22)	10.98 (1.7)	<0.05
Diagnostic delay (months)	16.26 (12.91)	17.49 (21.52)	0.66
Time since onset (months)	25.40 (19.30)	41.17 (149.2)	0.30
ΔFRS	0.65 (0.66)	0.56 (0.47)	0.38
Site of onset (Bulbar)	4 (16.67)	1 (9.09)	1.00
**Diagnosis**			
ALS/PLS/ALS-bvFTD	49/5/1	93/5/2	0.70

ALS, amyotrophic lateral sclerosis; ALSFRS-R, ALS functional rating scale-revised; BMI, body mass index; bvFTD, behavioural-variant frontotemporal dementia; PLS, primary lateral sclerosis; PBP, progressive bulbar palsy; CNAQ, Council on Nutrition Appetite Questionnaire; ΔFRS, decline in the ALSFRS-R (points/month since date of symptom onset).

^a^Data presented as mean (SD) for continuous variables or *n* counts (%) for categorical variables.

^b^Student *t*-tests with Welch's correction is conducted for continuous variables. Chi-squared tests are conducted for categorical variables if all expected numbers are five or greater. If this condition is not met, a Fisher's exact test is used instead.

^c^VAS measures only apply to participants who completed the imaging assessment.

^d^Four patients with a reported loss of appetite and one patient with a reported intact appetite had no metabolic data available.

### Associations between metabolism, appetite, weight loss and body composition

**Figure 3:**
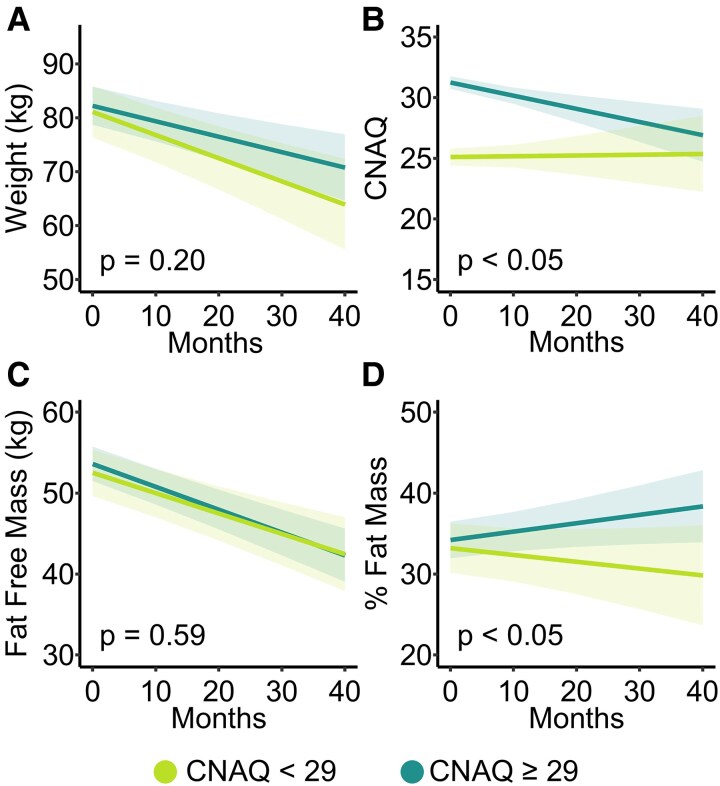
**Longitudinal measures in body composition of plwMND.** Changes in (**A**) weight (**B**) CNAQ (**C**) Fat-Free Mass and (**D**) Fat Mass in patients with a loss of appetite (CNAQ <29; *N* = 55) versus patients with intact appetite (CNAQ ≥ 29; *N* = 100). Longitudinal measures were analysed using linear mixed effects modelling.

For the assessment of change in anthropometric measures, we considered the effects of appetite on body weight and composition during follow-up (Fig. 3). We then compared outcomes from individuals with a baseline reported loss of appetite to those with a reported intact appetite. Weight and fat-free mass declined across all patients during follow-up, and there were no differences in the overall rate of decline between patients with intact versus loss of appetite. However, when considering the change in % fat mass (a proxy for overall nutritional status), we found that % fat mass tended to increase in patients with an intact appetite, and patients with a loss of appetite experienced a loss of % fat mass (*P* < 0.05). Overall, we observed a significant decrease in CNAQ scores when considering all patients with MND; however, rates of decline in appetite were greater in patients with intact appetite at study inclusion (*P* < 0.05). Similar observations were made in patients that progressed to imaging studies; patients with a loss of appetite tended to lose % fat mass, while patients with an intact appetite tended to increase in % fat mass (*P* < 0.05); however, CNAQ scores similarly decreased with time in both groups [*P* = 0.80; (Supplementary Fig. 1)]. We note that being hypermetabolic did not impact body weight (*P* = 0.33) and % fat mass (*P* = 0.57) change over the course of the disease.

### fMRI response to visual stimuli of non-food and food items

**Figure 4:**
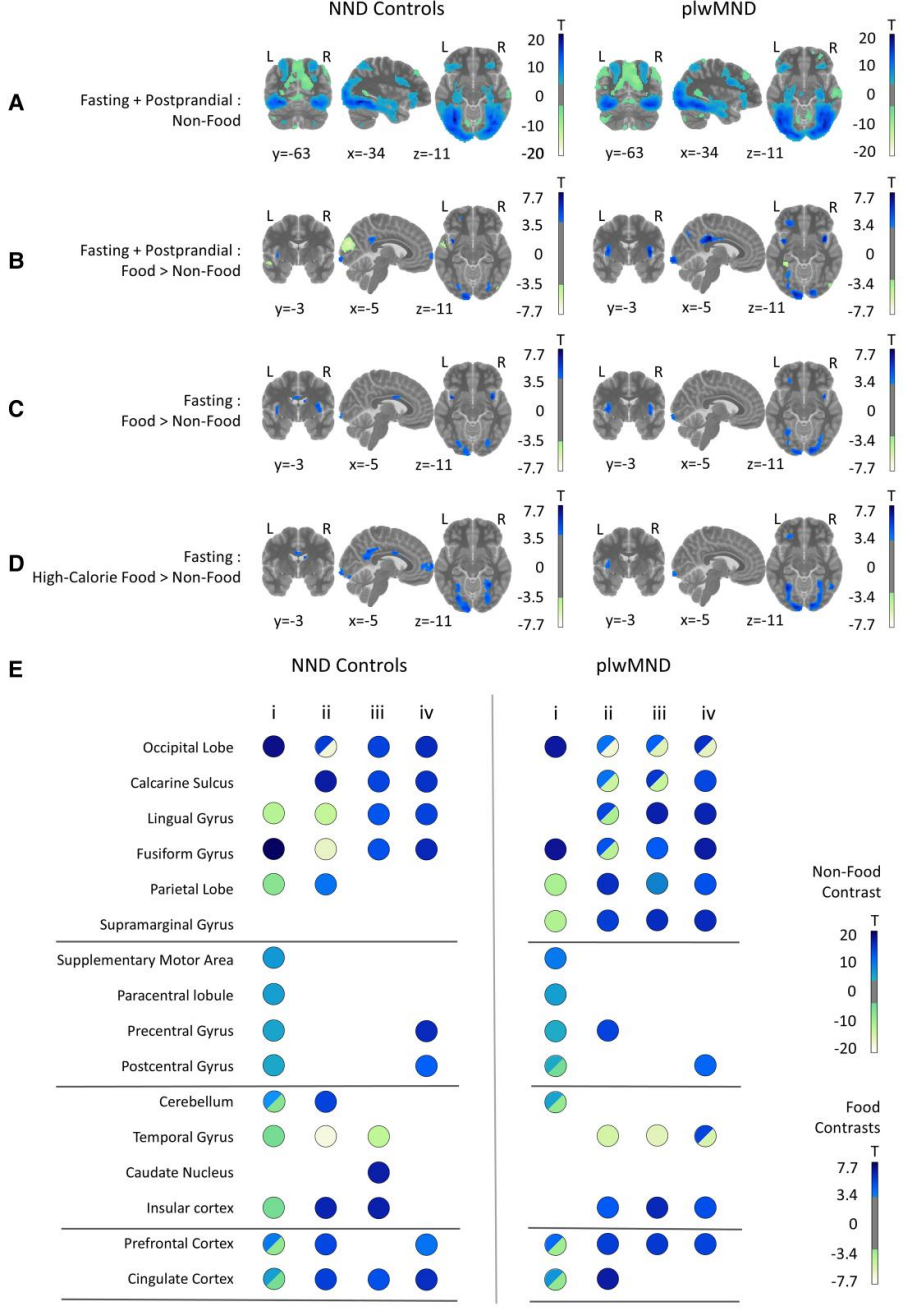
**BOLD response for visual stimuli of non-food and food items.** Contrasts visualized for NND controls (*N* = 23) and plwMND (*N* = 35) are (**A**) non-food items regardless of satiated state, (**B**) food items compared with non-food items regardless of satiated state, (**C**) food items compared with non-food items under a fasting state, (**D**) high-calorie food items compared with non-food items under a fasting state. Colour bars in (**A–D**) are thresholded at *T* = ± 3.35 (*P* = 0.001) for plwMND and *T* = ± 3.50 (*P* = 0.001) for NND controls. Contrasts were conducted using a one-sample or two-sample *t*-test. Only significant clusters (*P* < 0.05, corrected) are shown. (**E**) Cluster summary for each structure of interest for contrasts, in panels (**A–D**), labelled i–iv respectively. Structures with positive and negative clusters are represented with duo-toned circles. Colour bars in (**E**) are thresholded at *T* = ±3.35.

To ascertain the brain regions associated with altered responses to food cues in plwMND, we first sought to determine a baseline response to the visualisation of non-food items. To identify brain regions that respond specifically to food cues, we conducted several food contrasts against non-food stimuli under a fasted condition and post-prandial condition. BOLD responses of contrasts between visual stimuli of non-food and food items in fasting and following the consumption of the liquid meal are visualized as overlays in (Fig. 4A–D): non-food items (Fig. 4A), food > non-food (Fig. 4B), food > non-food under a fasting state (Fig. 4C), and high-calorie food > non-food under a fasting state (Fig. 4D). A summary of significant responses is provided in (Fig. 4E). Full descriptions of significant clusters are presented in (Supplementary Tables 3–10).

When shown images of non-food items, plwMND and NND controls had similar BOLD responses in areas consistent with object visualisation (Fig. 4A and Supplementary Tables 3 and 4). Both cohorts had significant positive BOLD responses spanning the visual processing centres—the inferior occipital lobe and fusiform gyrus, and negative responses in the parietal lobe. Significant clusters were observed in motor and somatosensory areas, and positive and negative clusters were observed in the cerebellum, prefrontal cortex and cingulate across both cohorts.

When presented with visual stimuli of food, contrasted against non-food items (Fig. 4B and Supplementary Tables 5 and 6), we found similar responses between plwMND and NND controls. Unlike stimuli of non-food items, we found positive and negative responses in the occipital lobe, calcarine sulcus, lingual gyrus, and fusiform gyrus, and positive responses in the parietal lobe. In plwMND, the supramarginal and precentral gyri had positive responses, whereas the cerebellum had a positive response in NND controls. Additionally, we saw negative clusters in the temporal gyrus in both cohorts, and positive responses in the insular, prefrontal, and cingulate cortices.

Following from previous fMRI food studies,^13^ we considered the BOLD response under the fasting condition for stimuli of food items and high-calorie food items. Under fasting conditions, the visual stimuli of either food or only high-calorie foods (Fig. 4C–D and Supplementary Tables 7–10) resulted in a positive response in the occipital lobe, calcarine sulcus and cingulate for NND controls, and positive and negative responses for plwMND. Positive responses were also recorded in the lingual and fusiform gyri for both cohorts, and both the parietal lobe and supramarginal gyrus had a positive response in plwMND. When visualising only high-calorie food items, we saw a positive response in the precentral gyrus for NND controls, while both cohorts registered positive clusters in the post-central gyrus. Positive and negative responses were also recorded in the temporal gyrus in plwMND. Finally, we saw broad positive responses in the insular, and prefrontal cortices of both cohorts.

### BOLD response relative to the provision of a liquid meal and measures of appetite

**Figure 5:**
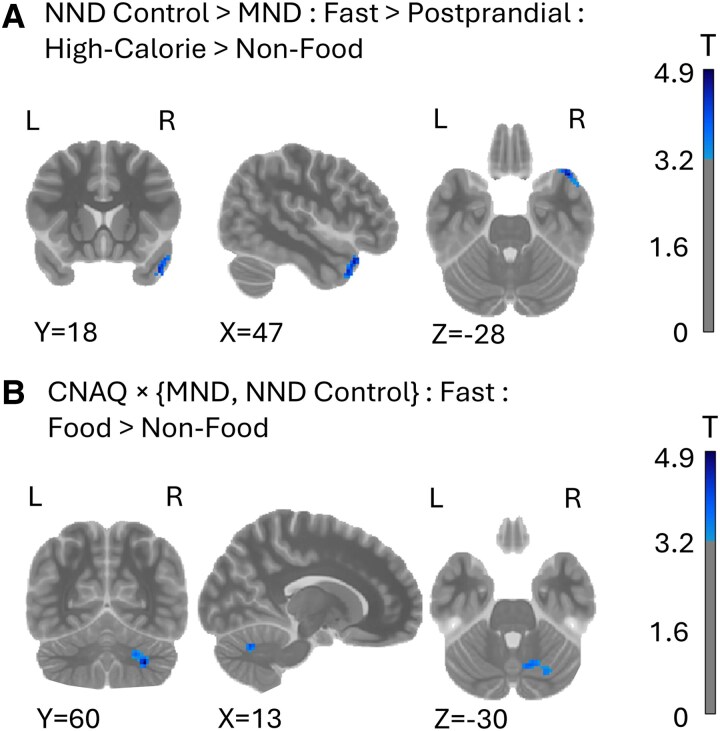
**BOLD response relative to the provision of the liquid meal and to measures of appetite.** (**A**) Significant cluster in the right temporal pole in NND control participants (*N* = 23) compared with plwMND (*N* = 35) when shown images of high-calorie foods versus non-food items under the fasted state compared with the post-prandial state. (**B**) Significant cluster in the right cerebellum nuclei indicating an interaction of measures of appetite (CNAQ scores) with disease status, when shown images of food items under a fasted condition. Colour bars for both panels are thresholded at *N* = ± 3.24 (*P* = 0.001). Contrasts were conducted using a two-sample *t*-test. Only significant clusters (*P* < 0.05, corrected) are shown.

To investigate the effect of ingestion of the liquid meal, we created the following contrast: Fasting (High-calorie > Non-Food) > Post-prandial (High-calorie > Non-Food). On the second-level, we further contrasted the BOLD response between plwMND and NND controls. When shown images of high-calorie foods under the fasting condition compared with the post-prandial state, plwMND had significantly decreased activity compared with NND controls in the right temporal pole (Fig. 5A; *P* = 0.021). The cluster spans the mid and superior temporal pole (Supplementary Table 11).

Finally, we considered how participants’ general state of appetite is associated with functional areas of the brain, and whether this association differs between plwMND and NND controls. We included CNAQ as part of the second-level design, and coded contrast to identify an interaction effect of the CNAQ score between the two cohorts. Results revealed a significant response in the right cerebellum (Fig. 5B; *P* = 0.001). This cluster covered 128 voxels in and around the cerebellar nuclei (Supplementary Table 11).

## Discussion

Loss of appetite is observed in patients with MND. It remains unclear how this impacts body weight regulation, and whether this occurs in response to widespread deficits in central regulation of appetite control. Conducting measures of appetite, metabolism and body weight and composition in 275 patients and controls, we show that loss of appetite, and not hypermetabolism, contributes to weight loss, and specifically loss of fat-free mass in plwMND. When considering potential mechanisms of appetite loss, we note that central responses to visual stimuli of food remain mostly intact in patients; however, some functional changes in brain areas outside of the hypothalamus could explain the loss of appetite in some plwMND. Specifically, we note that the temporal pole and cerebellum may be implicated in the loss of appetite, and thus we identify these brain regions as areas of interest for studies that aim to improve understanding of contributors of non-motor deficits of the disease. This is an important step towards an improved understanding of factors that impact quality of life, and thus the development of strategies aimed at improving outcomes for patients with MND.

Several reports have highlighted loss of appetite in MND as being of clinical concern.^4^ While associated with a reduction in energy intake,^31^ we demonstrate that loss of appetite contributes to weight loss specifically due to the progressive depletion of fat stores. We noted, also, no difference in prevalence of hypermetabolism between patients with intact versus loss of appetite, and so infer that lower appetite, and not hypermetabolism alone, predisposed these patients to negative energy balance. To confirm this, additional longitudinal studies that investigate the relationship between appetite, metabolism and body weight regulation are needed. Of interest, we found that appetite scores declined significantly during follow-up in patients with intact appetite at study inclusion, suggesting that most patients may be at risk of loss of appetite as the disease progresses. Collectively, these observations highlight a need to consider initial and regular assessments of appetite in patients as part of ongoing strategies to provide optimal dietary support to help patients meet their energy needs. Whether this will impact disease outcomes should also be considered. While we found that rates of disease progression (ΔFRS) were similar between patients with loss versus intact appetite at study inclusion, we are unable to investigate whether loss of appetite and the resulting weight loss will impact subsequent progression. Our cohort includes patients with slower progressing forms of the disease, including those with PLS. Additional studies are needed that specifically interrogate the impact of loss of appetite on functional decline and survival. Instead, we next considered factors thought to contribute to loss of appetite, and specifically the dysfunction of central appetite control as a contributing mechanism.

In general, plwMND and NND controls viewed images of non-food and food items similarly under a fasted state, and with fasting and post-prandial states combined. When shown images of non-food and food items, both cohorts had clusters in areas of the brain that are associated with object recognition, including the ventral stream, and the inferior occipital and fusiform gyrus. We observed clusters in the prefrontal cortex and the precentral gyrus, responses consistent with fMRI studies that present visual stimuli of neutral non-food images.^32^ We additionally identified clusters in the cingulate, an area of the brain shown to facilitate the processing of visual stimuli.^13^ Moreover, while generally associated with the regulation of fine motor control, clusters in the cerebellum in plwMND and NND controls are consistent with an emerging understanding of the cerebellum's role in sensory perception.^33^ Collectively, these observations demonstrate intact neural pathways involved in visual processing across this cohort of plwMND and NND controls.

Building on established research strategies for identifying central appetite mechanisms,^13^ we investigated responses to visual stimuli of high-calorie food cues. Prior studies have implicated the orbitofrontal cortex, left amygdala, insula, superior parietal lobe, and fusiform gyrus in the viewing of high-calorie foods.^13^ Our findings align with several studies, including the orbitofrontal cortex in reward encoding, and the contribution of the cingulum for decision making.^12,13^ Importantly, overall responses to visual stimuli of food were not statistically different between plwMND and NND controls, demonstrating that visual processing of food cues was generally intact across the whole cohort of patients.

The prandial status of an individual, whether fasted or fed, significantly impacts the appetitive brain network.^34^ When considering changes between the fasted and post-prandial state, we note a significant cluster in the right temporal pole in NND controls when compared with plwMND. While not traditionally considered to be involved in appetite regulation, some reports have highlighted a role for this area specifically in changes in appetite following the consumption of a meal. For example, the temporal pole was identified as a potentially critical component of the brain network that mediates self-regulation of food intake, especially during brief periods of increased restraint following a meal.^35^ The involvement of the temporal pole in appetite control could be considered ancillary, as it is included in a larger network thought to integrate multisensory stimuli and influence socio-emotional functions as well as visual cognition and memory.^36,37^ This is especially relevant when considering MND as part of a broader spectrum of neurodegenerative diseases. Up to 53% of plwMND present with signs of cognitive and/or behavioural involvement, which is associated with more severe cortical thinning,^38^ and changes in functional connectivity of the temporal pole.^39^ Importantly, 5–10% of these individuals may receive an eventual diagnosis of concomitant bvFTD,^19^ and bvFTD and ALS are considered to exist on a disease spectrum. Alterations in appetite are one of the key differences between the two extremes of this continuum^40^; individuals with bvFTD may experience an increase in caloric intake and preference for sweet foods, leading to an increase in BMI. In contrast, individuals with ALS may lose their appetite resulting in a greater loss of fat mass and BMI.^40^ While not directly assessed, inclusion of study participants across this spectrum may have contributed to altered responses in the temporal pole. We note that some study participants presented with mild cognitive deficits, and one participant had an eventual final diagnosis of ALS-bvFTD. Appetitive responses from these participants were mixed, and we found no clear association between their presentation and reports of appetite loss.

Although the cerebellum is not traditionally associated with the regulation of appetite, it has been shown to contribute to the processing of visual cues related to food.^41^ Moreover, altered cerebellar responses to food portions are proposed to contribute to the risk of overeating and weight gain.^42^ We found a reduced association between the cerebellum and the presentation of visual stimuli of food in plwMND. The cluster identified in this study is located around the deep cerebellar nuclei. These nuclei are associated with sensorimotor processing and food-motivated reward and form part of a larger functional network that includes sensorimotor areas, the hypothalamus and the striatum.^33^ Studies have also demonstrated consistent reductions in cerebellar grey matter volume and pathology associated with altered states of appetite,^43^ as well as anorexia nervosa,^44^ and obesity.^45^

Cerebellar pathology is reported in MND; however, its contribution to the pathophysiology of the disease remains unclear. Studies have found decreases in cerebellar grey matter volume in plwMND,^46^ as well as changes in white matter diffusivity.^47^ Functional studies show increases in functional activity, particularly during motor tasks, which has been suggested to play a compensatory role in MND.^48^ While the neuroplastic compensation of the cerebellum in MND remains under investigation, its established role in motor tasks and function suggests that changes in the cerebellum may also influence its involvement in regulating primal emotions.^49^ Indeed, cerebellar pathology is suspected to contribute to pseudobulbar affect and behavioural dysfunction in MND.^47^ Findings highlight a complex interplay between cerebellar pathology and appetite regulation in plwMND, suggesting that cerebellar changes may underpin not only compensatory mechanisms to motor deficits in MND, but also significant behavioural and emotional dysfunctions, including alterations in appetite.

While offering new insights into changes in appetite and appetite regulation in MND, we note several study limitations. The involvement of the temporal pole and cerebellum suggests a broader engagement of structures related to behaviour and emotions. We note, however, that few studies document the impact of psychosocial factors on appetite control in MND, and thus, the extent of these on appetite in MND remains unknown. Lower CNAQ scores were previously observed in plwMND with higher levels of anxiety and depression,^50^ suggesting that anxiety and depression likely contributes to loss of appetite in some plwMND. While plwMND with loss of appetite scored lower for question 8 of the CNAQ (on mood), we did not specifically interrogate the impact of anxiety and depression on appetite. Therefore, additional studies are needed to understand the link between biological and psychosocial outcomes, and their impact on patients. Furthermore, while we prioritized assessment of patients with a diagnosis of MND, we did not specifically include patients across the ALS-bvFTD spectrum, despite the significant variations in appetite changes observed across the spectrum.^40^ Thus, we were unable to address the impact of significant cognitive decline on appetite control in patients across the spectrum. We encourage future research to explore differences in functional responses across the disease spectrum, particularly in the temporal pole, which may yield novel insights into factors contributing to loss of appetite in plwMND with cognitive involvement.

We document the impact of loss of appetite on body weight and composition change and employed task-based fMRI to explore functional changes in response to visual food stimuli under fasting and post-prandial conditions. We demonstrate that loss of appetite impacts body weight regulation, and specifically the loss of body fat mass, and note worsening appetite with disease progression. While results demonstrate mostly intact central responses to visual food stimuli in patients with MND, we note several brain areas that could contribute to appetite loss. As such, we encourage a shift from current paradigms and the traditional focus on the hypothalamus to encompass a wider network of brain structures implicated in behaviour and emotion. More intensive investigations into how MND broadly impacts brain function are crucial to fully understanding the spectrum of MND pathology and its impact on non-motor symptoms of the disease.

## Supplementary Material

fcaf111_Supplementary_Data

## Data Availability

Deidentified imaging data and code from this study are available at https://doi.org/10.18112/openneuro.ds005874.v1.0.0. Additional data are available upon request from the corresponding author.

## References

[1] Hardiman O, Al-Chalabi A, Chio A, et al Amyotrophic lateral sclerosis. Nat Rev Dis Primers. 2017;3:17071. 28980624 10.1038/nrdp.2017.71

[2] Ludolph A, Dupuis L, Kasarskis E, Steyn F, Ngo S, McDermott C. Nutritional and metabolic factors in amyotrophic lateral sclerosis. Nat Rev Neurol. 2023;19(9):511–524. 37500993 10.1038/s41582-023-00845-8

[3] Shojaie A, Al Khleifat A, Sarraf P, Al-Chalabi A. Analysis of non-motor symptoms in amyotrophic lateral sclerosis. Amyotroph Lateral Scler Frontotemporal Degener. 2024;25(3–4):237–241. 37981575 10.1080/21678421.2023.2280618 PMC11238730

[4] Sarmet M, Kabani A, Maragakis NJ, Mehta AK. Appetite and quality of life in amyotrophic lateral sclerosis: A scoping review. Muscle Nerve. 2022;66(6):653–660. 35986916 10.1002/mus.27694

[5] Ngo ST, van Eijk RPA, Chachay V, et al Loss of appetite is associated with a loss of weight and fat mass in patients with amyotrophic lateral sclerosis. Amyotroph Lateral Scler Frontotemporal Degener. 2019;20(7–8):497–505. 31144522 10.1080/21678421.2019.1621346

[6] Howe SL, Holdom CJ, McCombe PA, et al Associations of postprandial ghrelin, liver-expressed antimicrobial peptide 2 and leptin levels with body composition, disease progression and survival in patients with amyotrophic lateral sclerosis. Eur J Neurol. 2024;31(1):e16052. 37658515 10.1111/ene.16052 PMC10840749

[7] Chang J, Shaw TB, Holdom CJ, et al Lower hypothalamic volume with lower body mass index is associated with shorter survival in patients with amyotrophic lateral sclerosis. Eur J Neurol. 2023;30(1):57–68. 36214080 10.1111/ene.15589 PMC10099625

[8] Gabery S, Ahmed RM, Caga J, Kiernan MC, Halliday GM, Petersén Å. Loss of the metabolism and sleep regulating neuronal populations expressing orexin and oxytocin in the hypothalamus in amyotrophic lateral sclerosis. Neuropathol Appl Neurobiol. 2021;47(7):979–989. 33755993 10.1111/nan.12709

[9] Michielsen A, van Veenhuijzen K, Janse van Mantgem MR, et al Association between hypothalamic volume and metabolism, cognition, and behavior in patients with amyotrophic lateral sclerosis. Neurology. 2024;103(2):e209603. 38875517 10.1212/WNL.0000000000209603 PMC11244736

[10] Bolborea M, Vercruysse P, Daria T, et al Loss of hypothalamic MCH decreases food intake in amyotrophic lateral sclerosis. Acta Neuropathol. 2023;145(6):773–791. 37058170 10.1007/s00401-023-02569-x PMC10175407

[11] Ahmed RM, Irish M, Henning E, et al Assessment of eating behavior disturbance and associated neural networks in frontotemporal dementia. JAMA Neurol. 2016;73(3):282–290. 26810632 10.1001/jamaneurol.2015.4478

[12] Dagher A . Functional brain imaging of appetite. Trends Endocrinol Metab. 2012;23(5):250–260. 22483361 10.1016/j.tem.2012.02.009

[13] Zheng L, Miao M, Gan Y. A systematic and meta-analytic review on the neural correlates of viewing high- and low-calorie foods among normal-weight adults. Neurosci Biobehav Rev. 2022;138:104721. 35667634 10.1016/j.neubiorev.2022.104721

[14] Sergi G, De Rui M, Coin A, Inelmen EM, Manzato E. Weight loss and Alzheimer's disease: Temporal and aetiologic connections. Proc Nutr Soc. 2013;72(1):160–165. 23110988 10.1017/S0029665112002753

[15] Ismail Z, Herrmann N, Rothenburg LS, et al A functional neuroimaging study of appetite loss in Alzheimer's disease. J Neurol Sci. 2008;271(1–2):97–103. 18495162 10.1016/j.jns.2008.03.023

[16] Ahmed RM, Tse NY, Chen Y, et al Neural correlates of fat preference in frontotemporal dementia: Translating insights from the obesity literature. Ann Clin Transl Neurol. 2021;8(6):1318–1329. 33973740 10.1002/acn3.51369 PMC8164857

[17] Brettschneider J, Del Tredici K, Toledo JB, et al Stages of pTDP-43 pathology in amyotrophic lateral sclerosis. Ann Neurol. 2013;74(1):20–38. 23686809 10.1002/ana.23937 PMC3785076

[18] Chipika RH, Christidi F, Finegan E, et al Amygdala pathology in amyotrophic lateral sclerosis and primary lateral sclerosis. J Neurol Sci. 2020;417:117039. 32713609 10.1016/j.jns.2020.117039

[19] Strong MJ, Abrahams S, Goldstein LH, et al Amyotrophic lateral sclerosis—Frontotemporal spectrum disorder (ALS-FTSD): Revised diagnostic criteria. Amyotroph Lateral Scler Frontotemporal Degener. 2017;18(3–4):153–174. 28054827 10.1080/21678421.2016.1267768 PMC7409990

[20] Holdom CJ, Janse van Mantgem MR, He J, et al Variation in resting metabolic rate affects identification of metabolic change in geographically distinct cohorts of patients with ALS. Neurology. 2024;102(5):e208117. 38350046 10.1212/WNL.0000000000208117

[21] Steyn FJ, Ioannides ZA, van Eijk RPA, et al Hypermetabolism in ALS is associated with greater functional decline and shorter survival. J Neurol Neurosurg Psychiatry. 2018;89(10):1016. 29706605 10.1136/jnnp-2017-317887 PMC6166607

[22] Siri WE . Body composition from fluid spaces and density: Analysis of methods. Techniques for measuring body composition. National Academy of Sciences, National Research Council; 1961:223–224.

[23] Wilson MM, Thomas DR, Rubenstein LZ, et al Appetite assessment: Simple appetite questionnaire predicts weight loss in community-dwelling adults and nursing home residents. Am J Clin Nutr. 2005;82(5):1074–1081. 16280441 10.1093/ajcn/82.5.1074

[24] Holm T, Maier A, Wicks P, et al Severe loss of appetite in amyotrophic lateral sclerosis patients: Online self-assessment study. Interact J Med Res. 2013;2(1):e8. 23608722 10.2196/ijmr.2463 PMC3632382

[25] Cedarbaum JM, Stambler N, Malta E, et al The ALSFRS-R: A revised ALS functional rating scale that incorporates assessments of respiratory function. BDNF ALS Study Group (Phase III). J Neurol Sci. 1999;169(1–2):13–21. 10540002 10.1016/s0022-510x(99)00210-5

[26] Flint A, Raben A, Blundell JE, Astrup A. Reproducibility, power and validity of visual analogue scales in assessment of appetite sensations in single test meal studies. Int J Obes Relat Metab Disord. 2000;24(1):38–48. 10702749 10.1038/sj.ijo.0801083

[27] Blechert J, Meule A, Busch NA, Ohla K. Food-pics: An image database for experimental research on eating and appetite. Front Psychol. 2014;5:617. 25009514 10.3389/fpsyg.2014.00617 PMC4067906

[28] Esteban O, Markiewicz CJ, Blair RW, et al fMRIPrep: A robust preprocessing pipeline for functional MRI. Nat Methods. 2019;16(1):111–116. 30532080 10.1038/s41592-018-0235-4 PMC6319393

[29] Tzourio-Mazoyer N, Landeau B, Papathanassiou D, et al Automated anatomical labeling of activations in SPM using a macroscopic anatomical parcellation of the MNI MRI single-subject brain. Neuroimage. 2002;15(1):273–289. 11771995 10.1006/nimg.2001.0978

[30] nilearn. Version 0.10.2. https://github.com/nilearn/nilearn

[31] Mezoian T, Belt E, Garry J, et al Loss of appetite in amyotrophic lateral sclerosis is associated with weight loss and decreased calorie consumption independent of dysphagia. Muscle Nerve. 2020;61(2):230–234. 31650547 10.1002/mus.26749

[32] Pati D, O'Boyle M, Amor C, Hou J, Valipoor S, Fang D. Neural correlates of nature stimuli: An FMRI study. Herd. 2014;7(2):9–28. 10.1177/193758671400700202 24554354

[33] Adamaszek M, D'Agata F, Ferrucci R, et al Consensus paper: Cerebellum and emotion. Cerebellum. 2017;16(2):552–576. 27485952 10.1007/s12311-016-0815-8

[34] Goldstone AP, Prechtl de Hernandez CG, Beaver JD, et al Fasting biases brain reward systems towards high-calorie foods. Eur J Neurosci. 2009;30(8):1625–1635. 19811532 10.1111/j.1460-9568.2009.06949.x

[35] Paolini BM, Laurienti PJ, Norris J, Rejeski WJ. Meal replacement: Calming the hot-state brain network of appetite. Front Psychol. 2014;5:249. 24723901 10.3389/fpsyg.2014.00249 PMC3971177

[36] Herlin B, Navarro V, Dupont S. The temporal pole: From anatomy to function-A literature appraisal. J Chem Neuroanat. 2021;113:101925. 33582250 10.1016/j.jchemneu.2021.101925

[37] Olson IR, Plotzker A, Ezzyat Y. The Enigmatic temporal pole: A review of findings on social and emotional processing. Brain. 2007;130(Pt 7):1718–1731. 17392317 10.1093/brain/awm052

[38] Agosta F, Ferraro PM, Riva N, et al Structural brain correlates of cognitive and behavioral impairment in MND. Hum Brain Mapp. 2016;37(4):1614–1626. 26833930 10.1002/hbm.23124 PMC6867462

[39] Loewe K, Machts J, Kaufmann J, et al Widespread temporo-occipital lobe dysfunction in amyotrophic lateral sclerosis. Sci Rep. 2017;7:40252. 28067298 10.1038/srep40252 PMC5220336

[40] Ahmed RM, Irish M, Piguet O, et al Amyotrophic lateral sclerosis and frontotemporal dementia: Distinct and overlapping changes in eating behaviour and metabolism. Lancet Neurol. 2016;15(3):332–342. 26822748 10.1016/S1474-4422(15)00380-4

[41] van der Laan LN, de Ridder DT, Viergever MA, Smeets PA. The first taste is always with the eyes: A meta-analysis on the neural correlates of processing visual food cues. Neuroimage. 2011;55(1):296–303. 21111829 10.1016/j.neuroimage.2010.11.055

[42] Fuchs BA, Pearce AL, Rolls BJ, et al The cerebellar response to visual portion size cues is associated with the portion size effect in children. Nutrients. 2024;16(5):738. 38474866 10.3390/nu16050738 PMC10933954

[43] Sader M, Waiter GD, Williams JHG. The cerebellum plays more than one role in the dysregulation of appetite: Review of structural evidence from typical and eating disorder populations. Brain Behav. 2023;13(12):e3286. 37830247 10.1002/brb3.3286 PMC10726807

[44] Tose K, Takamura T, Isobe M, et al Systematic reduction of gray matter volume in anorexia nervosa, but relative enlargement with clinical symptoms in the prefrontal and posterior insular cortices: A multicenter neuroimaging study. Mol Psychiatry. 2024;29(4):891–901. 38246936 10.1038/s41380-023-02378-4 PMC11176065

[45] Herrmann MJ, Tesar AK, Beier J, Berg M, Warrings B. Grey matter alterations in obesity: A meta-analysis of whole-brain studies. Obes Rev. 2019;20(3):464–471. 30537231 10.1111/obr.12799

[46] Christidi F, Karavasilis E, Velonakis G, et al Motor and extra-motor gray matter integrity may underlie neurophysiologic parameters of motor function in amyotrophic lateral sclerosis: A combined voxel-based morphometry and transcranial stimulation study. Brain Imaging Behav. 2018;12(6):1730–1741. 29417490 10.1007/s11682-018-9841-0

[47] Bede P, Chipika RH, Christidi F, et al Genotype-associated cerebellar profiles in ALS: Focal cerebellar pathology and cerebro-cerebellar connectivity alterations. J Neurol Neurosurg Psychiatry. 2021;92(11):1197–1205. 34168085 10.1136/jnnp-2021-326854 PMC8522463

[48] Abidi M, de Marco G, Grami F, et al Neural correlates of motor imagery of gait in amyotrophic lateral sclerosis. J Magn Reson Imaging. 2021;53(1):223–233. 32896088 10.1002/jmri.27335

[49] Schutter D . The cerebellum in emotions and psychopathology. Routledge; 2020.

[50] Wang Y, Ye S, Chen L, Tang L, Fan D. Loss of appetite in patients with amyotrophic lateral sclerosis is associated with weight loss and anxiety/depression. Sci Rep. 2021;11(1):9119. 33907295 10.1038/s41598-021-88755-x PMC8079393

