# Supplementary material for: Appetite loss in patients with motor neuron disease: impact on weight loss and neural correlates of visual food cues
Source: Brain Commun. 2025 Mar 14;7(2):fcaf111. doi: 10.1093/braincomms/fcaf111 (PMC11938820; doi:10.1093/braincomms/fcaf111)
Supplement: fcaf111_Supplementary_Data [file fcaf111_supplementary_data.docx]

# Supplementary methods

## Imaging study protocol

All scans were acquired using a 3-Tesla Siemens Prisma scanner (Siemens Healthcare, Erlangen, Germany). T1-weighted structural scans were obtained in the first session from a 3-dimensional 1 mm^3^ isotropic MP2RAGE sequence (TR/TE/TIs/Flip Angles/FoV/Acquisition Time=5000ms/2.98ms/701ms,2500ms/4°,5°/256mm×240 mm/9m:02s).^1^ Functional scans were obtained from both sessions through a 2D gradient echo planar imaging sequence (TR/TE/Flip Angle/Acquisition Time =820ms/33ms/53°/11m:12s), with a voxel size of 2.4x×.4×2.4 mm, and with 60 slices that covers a 206mm field of view which includes the entire cortex and cerebellum. During scans, participants were equipped with a pulse oximeter and a breathing belt to record physiological activity.

## fMRI processing

Anatomical and functional processing was conducted in fMRIPrep 23.1.4.^2^ See below for the methods boilerplate from fMRIPrep. To account for physiological noise originating from respiration and cardiac activity, the PhysIO toolbox^3^ was used to process breathing belt and pulse oximetry data, creating eight regressors for respiration and six regressors for cardiac activity. Additionally, to account for excessive movement, any volumes exceeding a framewise displacement of 1 mm were censored in the resulting design matrix. Spatial smoothing was applied using an 8 mm full-width half-maximum Gaussian kernel.

## fMRI contrasts

Analysis was conducted using the Statistical Parameter Mapping 12 (SPM12) toolbox (Wellcome Centre for Human Neuroimaging, London UK; [www.fil.ion.ucl.ac.uk/spm/](http://www.fil.ion.ucl.ac.uk/spm/)).^4^ Regressors for the presentation of high-calorie food, low-calorie food, and non-food items were created based on their onset and duration times, which were then convolved with the haemodynamic response function. In addition, physiological noise regressors and motion parameters estimates from the image realignment were added to the design matrix. Voxel-wise parameter estimates were obtained for each participant, and a random effects group analysis was performed using the standard summary statistics approach implemented in SPM. The visualisation of contrasts was generated using the Python library, Nilearn.^5^

# References

1. Marques JP, Kober T, Krueger G, van der Zwaag W, Van de Moortele PF, Gruetter R. MP2RAGE, a self bias-field corrected sequence for improved segmentation and T1-mapping at high field. *Neuroimage*. Jan 15 2010;49(2):1271-81. doi:10.1016/j.neuroimage.2009.10.002

2. Esteban O, Markiewicz CJ, Blair RW, et al. fMRIPrep: a robust preprocessing pipeline for functional MRI. *Nat Methods*. Jan 2019;16(1):111-116. doi:10.1038/s41592-018-0235-4

3. Kasper L, Bollmann S, Diaconescu AO, et al. The PhysIO Toolbox for Modeling Physiological Noise in fMRI Data. *J Neurosci Methods*. Jan 30 2017;276:56-72. doi:10.1016/j.jneumeth.2016.10.019

4. Penny WD, Friston KJ, Ashburner JT, Kiebel SJ, Nichols TE. *Statistical parametric mapping: the analysis of functional brain images*. Elsevier; 2007.

5. Abraham A, Pedregosa F, Eickenberg M, et al. Machine learning for neuroimaging with scikit-learn. *Front Neuroinform*. 2014;8:14. doi:10.3389/fninf.2014.00014

6. Blechert J, Meule A, Busch NA, Ohla K. Food-pics: an image database for experimental research on eating and appetite. *Front Psychol*. 2014;5:617. doi:10.3389/fpsyg.2014.00617

## fMRIPrep methods

### Copyright Waiver

The below boilerplate text was automatically generated by fMRIPrep with the express intention that users should copy and paste this text into their manuscripts *unchanged*. It is released under the [CC0](https://creativecommons.org/publicdomain/zero/1.0/) license.

### Anatomical data pre-processing

Preprocessing was performed using *fMRIPrep* 23.1.4 (Esteban et al. (2019); Esteban et al. (2018); RRID:SCR_016216), which is based on *Nipype* 1.8.6 (K. Gorgolewski et al. (2011); K. J. Gorgolewski et al. (2018); RRID:SCR_002502).

T1-weighted (T1w) images were found within the input BIDS dataset and a single randomly selected image was corrected for intensity non-uniformity (INU) with N4BiasFieldCorrection (Tustison et al. 2010), distributed with ANTs (version unknown) (Avants et al. 2008, RRID:SCR_004757), and used as T1w-reference throughout the workflow. The T1w-reference was skull-stripped with a *Nipype* implementation of the antsBrainExtraction.sh workflow (from ANTs), using OASIS30ANTs as target template. Brain tissue segmentation of cerebrospinal fluid (CSF), white-matter (WM) and gray-matter (GM) was performed on the brain-extracted T1w using fast (FSL (version unknown), RRID:SCR_002823, Zhang, Brady, and Smith 2001). Brain surfaces were reconstructed using recon-all (FreeSurfer 7.3.2, RRID:SCR_001847, Dale, Fischl, and Sereno 1999), and the brain mask estimated previously was refined with a custom variation of the method to reconcile ANTs-derived and FreeSurfer-derived segmentations of the cortical gray-matter of Mindboggle (RRID:SCR_002438, Klein et al. 2017). Volume-based spatial normalization to one standard space (MNI152NLin2009cAsym) was performed through nonlinear registration with antsRegistration (ANTs (version unknown)), using brain-extracted versions of both T1w reference and the T1w template. The following template was selected for spatial normalization and accessed with *TemplateFlow* (23.0.0, Ciric et al. 2022): *ICBM 152 Nonlinear Asymmetrical template version 2009c* [Fonov et al. (2009), RRID:SCR_008796; TemplateFlow ID: MNI152NLin2009cAsym].

### Preprocessing of B0 inhomogeneity mappings

A total of 4 fieldmaps were found available within the input BIDS structure for the participant that contributed the representative T1W image. A deformation field was estimated, based on *fMRIPrep*’s *fieldmap-less* approach, to correct for susceptibility distortions. The deformation field is that resulting from co-registering the EPI reference to the same-subject T1w-reference with its intensity inverted (Wang et al. 2017; Huntenburg 2014). Registration is performed with antsRegistration (ANTs – version unknown), and the process regularized by constraining deformation to be nonzero only along the phase-encoding direction and modulated with an average fieldmap template (Treiber et al. 2016).

### Functional data pre-processing

For each of the 4 BOLD runs found per subject (across all tasks and sessions), the following pre-processing was performed. First, a reference volume and its skull-stripped version were generated using a custom methodology of *fMRIPrep*. Head-motion parameters with respect to the BOLD reference (transformation matrices, and six corresponding rotation and translation parameters) were estimated before any spatiotemporal filtering using mcflirt (et FSL, Jenkinson al. 2002). The estimated *fieldmap* was then aligned with rigid-registration to the target EPI (echo-planar imaging) reference run. The field coefficients were mapped on to the reference EPI using the transform. BOLD runs were slice-time corrected to 0.36s (0.5 of slice acquisition range 0s-0.72s) using 3dTshift from AFNI (Cox and Hyde 1997, RRID:SCR_005927). The BOLD reference was then co-registered to the T1w reference using bbregister (FreeSurfer) which implements boundary-based registration (Greve and Fischl 2009). Co-registration was configured with twelve degrees of freedom to account for distortions remaining in the BOLD reference. Several confounding time-series were calculated based on the *preprocessed BOLD*: framewise displacement (FD), DVARS and three region-wise global signals. FD was computed using two formulations following Power (absolute sum of relative motions, Power et al. (2014)) and Jenkinson (relative root mean square displacement between affines, Jenkinson et al. (2002)). FD and DVARS were calculated for each functional run, both using their implementations in *Nipype* (following the definitions by Power et al. 2014). The three global signals were extracted within the CSF, the WM, and the whole-brain masks. Additionally, a set of physiological regressors were extracted to allow for component-based noise correction (*CompCor*, Behzadi et al. 2007). Principal components were estimated after high-pass filtering the pre-processed BOLD time-series (using a discrete cosine filter with 128s cut-off) for the two *CompCor* variants: temporal (tCompCor) and anatomical (aCompCor). tCompCor components were then calculated from the top 2% variable voxels within the brain mask. For aCompCor, three probabilistic masks (CSF, WM and combined CSF+WM) were generated in anatomical space. The implementation differs from that of Behzadi et al. in that instead of eroding the masks by 2 pixels on BOLD space, a mask of pixels that likely contain a volume fraction of GM is subtracted from the aCompCor masks. This mask is obtained by dilating a GM mask extracted from the FreeSurfer’s *aseg* segmentation, and ensures components are not extracted from voxels containing a minimal fraction of GM. Finally, these masks were resampled into BOLD space and binarized by thresholding at 0.99 (as in the original implementation). Components were also calculated separately within the WM and CSF masks. For each CompCor decomposition, the *k* components with the largest singular values were retained, such that the retained components’ time series were sufficient to explain 50 percent of variance across the nuisance mask (CSF, WM, combined, or temporal). The remaining components were dropped from consideration. The head-motion estimates calculated in the correction step were also placed within the corresponding confounds file. The confound time series derived from head motion estimates and global signals were expanded with the inclusion of temporal derivatives and quadratic terms for each (Satterthwaite et al. 2013). Frames that exceeded a threshold of 0.5 mm FD or 1.5 standardized DVARS were annotated as motion outliers. Additional nuisance timeseries were calculated by means of principal components analysis of the signal found within a thin band (*crown*) of voxels around the edge of the brain, as proposed by (Patriat, Reynolds, and Birn 2017). The BOLD time-series were resampled into standard space, generating a *preprocessed BOLD run in MNI152NLin2009cAsym space*. First, a reference volume and its skull-stripped version were generated using a custom methodology of *fMRIPrep*. All resamplings can be performed with *a single interpolation step* by composing all the pertinent transformations (i.e. head-motion transform matrices, susceptibility distortion correction when available, and co-registrations to anatomical and output spaces). Gridded (volumetric) resamplings were performed using antsApplyTransforms (ANTs), configured with Lanczos interpolation to minimize the smoothing effects of other kernels (Lanczos 1964). Non-gridded (surface) resamplings were performed using mri_vol2surf (FreeSurfer).

Many internal operations of *fMRIPrep* use *Nilearn* 0.10.1 (Abraham et al. 2014, RRID:SCR_001362), mostly within the functional processing workflow. For more details of the pipeline, see [the section corresponding to workflows in *fMRIPrep*’s documentation](https://fmriprep.readthedocs.io/en/latest/workflows.html).

### References

Abraham, Alexandre, Fabian Pedregosa, Michael Eickenberg, Philippe Gervais, Andreas Mueller, Jean Kossaifi, Alexandre Gramfort, Bertrand Thirion, and Gael Varoquaux. 2014. “Machine Learning for Neuroimaging with Scikit-Learn.” *Frontiers in Neuroinformatics* 8. <https://doi.org/10.3389/fninf.2014.00014>.

Avants, B. B., C. L. Epstein, M. Grossman, and J. C. Gee. 2008. “Symmetric Diffeomorphic Image Registration with Cross-Correlation: Evaluating Automated Labeling of Elderly and Neurodegenerative Brain.” *Medical Image Analysis* 12 (1): 26–41. <https://doi.org/10.1016/j.media.2007.06.004>.

Behzadi, Yashar, Khaled Restom, Joy Liau, and Thomas T. Liu. 2007. “A Component Based Noise Correction Method (CompCor) for BOLD and Perfusion Based fMRI.” *NeuroImage* 37 (1): 90–101. <https://doi.org/10.1016/j.neuroimage.2007.04.042>.

Ciric, R., William H. Thompson, R. Lorenz, M. Goncalves, E. MacNicol, C. J. Markiewicz, Y. O. Halchenko, et al. 2022. “TemplateFlow: FAIR-Sharing of Multi-Scale, Multi-Species Brain Models.” *Nature Methods* 19: 1568–71. <https://doi.org/10.1038/s41592-022-01681-2>.

Cox, Robert W., and James S. Hyde. 1997. “Software Tools for Analysis and Visualization of fMRI Data.” *NMR in Biomedicine* 10 (4-5): 171–78. [https://doi.org/10.1002/(SICI)1099-1492(199706/08)10:4/5<171::AID-NBM453>3.0.CO;2-L](https://doi.org/10.1002/(SICI)1099-1492(199706/08)10:4/5%3c171::AID-NBM453%3e3.0.CO;2-L).

Dale, Anders M., Bruce Fischl, and Martin I. Sereno. 1999. “Cortical Surface-Based Analysis: I. Segmentation and Surface Reconstruction.” *NeuroImage* 9 (2): 179–94. <https://doi.org/10.1006/nimg.1998.0395>.

Esteban, Oscar, Ross Blair, Christopher J. Markiewicz, Shoshana L. Berleant, Craig Moodie, Feilong Ma, Ayse Ilkay Isik, et al. 2018. “fMRIPrep 23.1.4.” *Software*. <https://doi.org/10.5281/zenodo.852659>.

Esteban, Oscar, Christopher Markiewicz, Ross W Blair, Craig Moodie, Ayse Ilkay Isik, Asier Erramuzpe Aliaga, James Kent, et al. 2019. “fMRIPrep: A Robust Preprocessing Pipeline for Functional MRI.” *Nature Methods* 16: 111–16. <https://doi.org/10.1038/s41592-018-0235-4>.

Fonov, VS, AC Evans, RC McKinstry, CR Almli, and DL Collins. 2009. “Unbiased Nonlinear Average Age-Appropriate Brain Templates from Birth to Adulthood.” *NeuroImage* 47, Supplement 1: S102. <https://doi.org/10.1016/S1053-8119(09)70884-5>.

Gorgolewski, K., C. D. Burns, C. Madison, D. Clark, Y. O. Halchenko, M. L. Waskom, and S. Ghosh. 2011. “Nipype: A Flexible, Lightweight and Extensible Neuroimaging Data Processing Framework in Python.” *Frontiers in Neuroinformatics* 5: 13. <https://doi.org/10.3389/fninf.2011.00013>.

Gorgolewski, Krzysztof J., Oscar Esteban, Christopher J. Markiewicz, Erik Ziegler, David Gage Ellis, Michael Philipp Notter, Dorota Jarecka, et al. 2018. “Nipype.” *Software*. <https://doi.org/10.5281/zenodo.596855>.

Greve, Douglas N, and Bruce Fischl. 2009. “Accurate and Robust Brain Image Alignment Using Boundary-Based Registration.” *NeuroImage* 48 (1): 63–72. <https://doi.org/10.1016/j.neuroimage.2009.06.060>.

Huntenburg, Julia M. 2014. “Evaluating Nonlinear Coregistration of BOLD EPI and T1w Images.” Master's Thesis, Berlin: Freie Universität. <http://hdl.handle.net/11858/00-001M-0000-002B-1CB5-A>.

Jenkinson, Mark, Peter Bannister, Michael Brady, and Stephen Smith. 2002. “Improved Optimization for the Robust and Accurate Linear Registration and Motion Correction of Brain Images.” *NeuroImage* 17 (2): 825–41. <https://doi.org/10.1006/nimg.2002.1132>.

Klein, Arno, Satrajit S. Ghosh, Forrest S. Bao, Joachim Giard, Yrjö Häme, Eliezer Stavsky, Noah Lee, et al. 2017. “Mindboggling Morphometry of Human Brains.” *PLOS Computational Biology* 13 (2): e1005350. <https://doi.org/10.1371/journal.pcbi.1005350>.

Lanczos, C. 1964. “Evaluation of Noisy Data.” *Journal of the Society for Industrial and Applied Mathematics Series B Numerical Analysis* 1 (1): 76–85. <https://doi.org/10.1137/0701007>.

Patriat, Rémi, Richard C. Reynolds, and Rasmus M. Birn. 2017. “An Improved Model of Motion-Related Signal Changes in fMRI.” *NeuroImage* 144, Part A (January): 74–82. <https://doi.org/10.1016/j.neuroimage.2016.08.051>.

Power, Jonathan D., Anish Mitra, Timothy O. Laumann, Abraham Z. Snyder, Bradley L. Schlaggar, and Steven E. Petersen. 2014. “Methods to Detect, Characterize, and Remove Motion Artifact in Resting State fMRI.” *NeuroImage* 84 (Supplement C): 320–41. <https://doi.org/10.1016/j.neuroimage.2013.08.048>.

Satterthwaite, Theodore D., Mark A. Elliott, Raphael T. Gerraty, Kosha Ruparel, James Loughead, Monica E. Calkins, Simon B. Eickhoff, et al. 2013. “An improved framework for confound regression and filtering for control of motion artifact in the preprocessing of resting-state functional connectivity data.” *NeuroImage* 64 (1): 240–56. <https://doi.org/10.1016/j.neuroimage.2012.08.052>.

Treiber, Jeffrey Mark, Nathan S. White, Tyler Christian Steed, Hauke Bartsch, Dominic Holland, Nikdokht Farid, Carrie R. McDonald, Bob S. Carter, Anders Martin Dale, and Clark C. Chen. 2016. “Characterization and Correction of Geometric Distortions in 814 Diffusion Weighted Images.” *PLOS ONE* 11 (3): e0152472. <https://doi.org/10.1371/journal.pone.0152472>.

Tustison, N. J., B. B. Avants, P. A. Cook, Y. Zheng, A. Egan, P. A. Yushkevich, and J. C. Gee. 2010. “N4ITK: Improved N3 Bias Correction.” *IEEE Transactions on Medical Imaging* 29 (6): 1310–20. <https://doi.org/10.1109/TMI.2010.2046908>.

Wang, Sijia, Daniel J. Peterson, J. C. Gatenby, Wenbin Li, Thomas J. Grabowski, and Tara M. Madhyastha. 2017. “Evaluation of Field Map and Nonlinear Registration Methods for Correction of Susceptibility Artifacts in Diffusion MRI.” *Frontiers in Neuroinformatics* 11. <https://doi.org/10.3389/fninf.2017.00017>.

Zhang, Y., M. Brady, and S. Smith. 2001. “Segmentation of Brain MR Images Through a Hidden Markov Random Field Model and the Expectation-Maximization Algorithm.” *IEEE Transactions on Medical Imaging* 20 (1): 45–57. <https://doi.org/10.1109/42.906424>.

# Supplementary figure

**
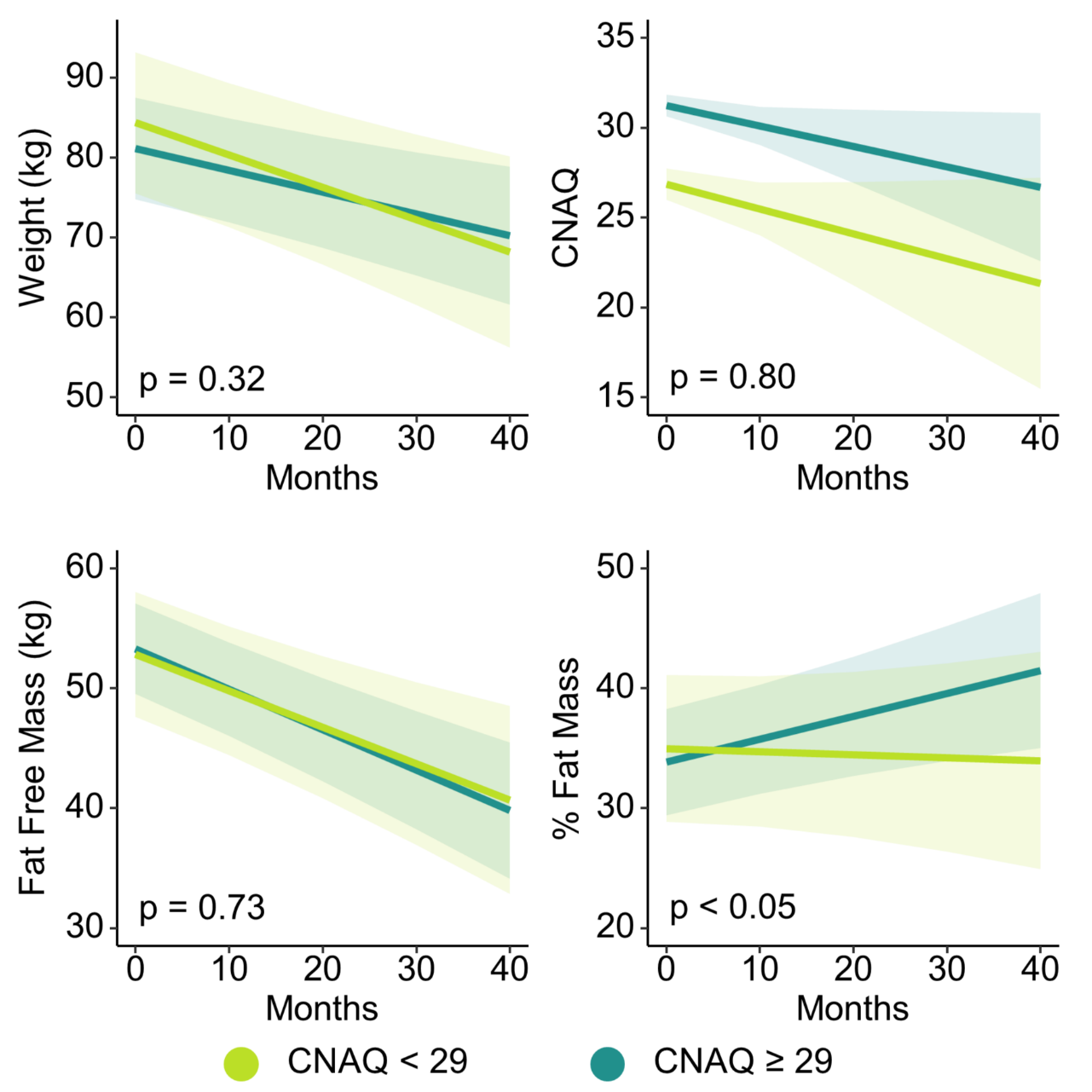
**

**Supplementary Figure 1 Longitudinal measures in body composition of plwMND in the imaging cohort.** Changes in **(A)** weight **(B)** CNAQ **(C)** Fat-Free Mass and **(D)** Fat Mass in patients with a loss of appetite (CNAQ < 29; N=11) versus patients with intact appetite (CNAQ ≥ 29;N=24). Longitudinal measures were analysed using linear mixed effects modelling.

# Supplementary tables

**Supplementary Table 1** Catalogue numbers and item descriptions from the FoodPics database selected and used in each image set.

| **Image Set 1** | |  | **Image Set 2** | |
| --- | --- | --- | --- | --- |
| **Image #** | **Item Description** |  | **Image #** | **Item Description** |
| 1 | cheesecake with cherry topping |  | 11 | cheese and cold meat platter |
| 2 | hamburger with bacon |  | 12 | ham sandwich |
| 3 | cheese burger, french fries and cola |  | 17 | cheese burger, french fries and cola |
| 6 | cheese cake, strawberries, crumbles |  | 23 | ham sandwich |
| 8 | snack mix |  | 34 | ham and cheese sandwich |
| 22 | french fries |  | 45 | Hamburger |
| 24 | ice cream sandwiches |  | 46 | french fries |
| 27 | opened chips bag |  | 47 | french fries and chicken drumsticks |
| 32 | pizza (with mushrooms) |  | 49 | chocolate popsicles |
| 48 | chocolate muffin |  | 60 | french fries |
| 49 | chocolate popsicles |  | 61 | pizza (salami) |
| 57 | ham sandwich with chips |  | 66 | croissants |
| 65 | cheese burger |  | 72 | sandwich roastbeef |
| 68 | doner kebab |  | 73 | Spaghetti Bolognese |
| 70 | cheese |  | 85 | Pizza (with salami) |
| 73 | Spaghetti Bolognese |  | 86 | cheese burger, french fries |
| 76 | spaghetti with tomato sauce |  | 87 | cheese burger |
| 77 | ham sandwich with chips |  | 91 | cheese burger |
| 81 | pizza slices (assorted) |  | 107 | chocolate cake |
| 86 | cheese burger, french fries |  | 112 | opened bar of chocolate with nuts |
| 87 | cheese burger |  | 118 | sundae (peach) |
| 93 | muffins |  | 120 | croissants |
| 97 | chocolate cake |  | 131 | pizza (veggie/cheese) |
| 108 | pizza (ham and mushrooms) |  | 144 | pasta bake |
| 111 | bar of chocolate with nuts |  | 145 | lasagna |
| 114 | croissants |  | 174 | chocolate pieces |
| 115 | sundae (with raspberries) |  | 176 | salami sausage |
| 130 | cake |  | 177 | cookies filled with chocolate cream |
| 135 | cake |  | 178 | German krapfen |
| 142 | pasta bake |  | 181 | bowl of muesli (granola) |
| 143 | pasta bake |  | 184 | chocolate croissants |
| 151 | lemon cake |  | 188 | doner kebab |
| 162 | chocolate cream cake |  | 194 | kiwi |
| 163 | chocolate cake |  | 195 | cucumber slice |
| 196 | salad plate |  | 199 | watermelon |
| 201 | salad plate |  | 202 | blueberries |
| 203 | wildberries mix |  | 206 | raspberries |
| 210 | raspberries |  | 207 | redcurrants |
| 211 | strawberries |  | 209 | wildberries mix |
| 214 | wildberries mix |  | 211 | strawberries |
| 215 | cucumber and carrot |  | 222 | strawberries |
| 232 | bowl of salad |  | 223 | wildberries mix |
| 252 | lettuce (iceberg) |  | 224 | wildberries mix |
| 253 | pickles |  | 225 | crisp read with cottage cheese |
| 260 | asparagus |  | 228 | bowl of salad |
| 261 | soybean sprouts |  | 229 | salad plate |
| 265 | zucchini |  | 234 | strawberries |
| 266 | green onion (shallot) |  | 243 | strawberries |
| 267 | cucumber with slices |  | 247 | green paprika peppers |
| 271 | parsley |  | 248 | blueberries |
| 278 | fennel |  | 255 | pomegranate |
| 280 | cherries |  | 258 | radishes |
| 286 | bar of chocolate |  | 262 | celery |
| 288 | carrots |  | 263 | mushrooms (brown) |
| 294 | popcorn |  | 264 | mushrooms (white) |
| 301 | roasted chicken |  | 272 | chives |
| 318 | fried sausage with roll |  | 273 | arugula |
| 323 | trout with potatoes, pan fried |  | 274 | spinach |
| 328 | fillet of pork, grilled |  | 277 | fennel |
| 332 | mixed salad |  | 280 | cherries |
| 335 | sugar snap pea |  | 284 | grapes |
| 338 | mixed licorice |  | 303 | cauliflower |
| 360 | artichoke |  | 304 | potato gratin |
| 362 | beans and carrots, cooked |  | 305 | peas, corn and potatos |
| 364 | green beans |  | 308 | salmon sushi |
| 369 | sushi roll with cucumber |  | 348 | rusk |
| 371 | sushi with salmon |  | 361 | carrots, cooked |
| 378 | ravioli |  | 366 | bagels with sesame and poppy seed |
| 380 | gherkin |  | 386 | papaya |
| 382 | gherkin, sliced |  | 400 | éclair |
| 384 | tortellini |  | 401 | red chilli |
| 394 | grape, whites |  | 405 | head of green lettuce |
| 398 | cherries |  | 407 | blackberries |
| 399 | cherry |  | 428 | artichoke |
| 403 | pizza with mushrooms, bell pepper |  | 429 | yellow bell pepper, sliced |
| 414 | mixed vegetables |  | 444 | green bell pepper |
| 417 | pea pod |  | 445 | yellow bell pepper |
| 432 | roman lettuce |  | 455 | spinach |
| 442 | red bell pepper |  | 458 | lemon 1, half |
| 445 | yellow bell pepper |  | 470 | pancakes with blueberries |
| 446 | tomato, sliced |  | 471 | French Toast |
| 448 | garden radish |  | 474 | croissant with butter and jam |
| 449 | garden radish |  | 485 | burger patty with french fries and salad |
| 453 | peach |  | 489 | pizza with salami |
| 460 | tomato |  | 491 | sundae |
| 467 | rasberry |  | 494 | pretzel |
| 473 | cheeseburger |  | 513 | olives |
| 479 | papaya |  | 516 | Edam cheese |
| 482 | mixed salad |  | 517 | cheese |
| 483 | pancake with fruits |  | 521 | rigatoni with vegetable |
| 486 | spare ribs, french fries and salad |  | 522 | vegetables |
| 492 | vanilla and chocolate icecream cone |  | 527 | tuna sandwiches |
| 493 | spare ribs, french fries and salad |  | 530 | strawberry, half |
| 496 | sausage |  | 531 | rasberries |
| 507 | waffle |  | 545 | shrimp |
| 509 | mixed vegetables |  | 551 | spaghetti with pesto |
| 520 | green chili |  | 552 | roast beef |
| 522 | vegetables |  | 557 | roast pork |
| 531 | rasberries |  | 562 | cutlet |
| 551 | spaghetti with pesto |  | 568 | salami, roasted ham and cheese |
| 1003 | flower bouquet |  | 1018 | paintbrushes |
| 1004 | shoe brush |  | 1019 | thumbtacks |
| 1005 | hair brush |  | 1021 | rose |
| 1010 | hammer |  | 1028 | tacker |
| 1014 | ladder |  | 1031 | telephone |
| 1015 | puncher |  | 1032 | clock (silver) |
| 1016 | nails |  | 1035 | books |
| 1020 | rose |  | 1037 | flower bouquet |
| 1022 | scissors |  | 1049 | shoe brushes |
| 1023 | screw |  | 1050 | chess men |
| 1025 | sun flower |  | 1112 | hand shovel |
| 1028 | tacker |  | 1134 | hammer |
| 1031 | telephone |  | 1139 | screw |
| 1033 | clock (schwarz) |  | 1140 | calculator |
| 1052 | shelf |  | 1152 | monthly planner |
| 1057 | checkerboard |  | 1153 | clipboard |
| 1077 | cake baking pan |  | 1157 | butterfly |
| 1087 | bag of LEGO figures |  | 1166 | butterfly 6 |
| 1096 | car rim |  | 1171 | elephant |
| 1111 | plastic cow figure |  | 1183 | fox |
| 1120 | burger carton |  | 1185 | cat |
| 1138 | cog wheel |  | 1186 | dog 4 |
| 1141 | book, closed |  | 1188 | shell |
| 1148 | phone |  | 1191 | butterfly 14 moth |
| 1149 | magnifying glass |  | 1192 | frog |
| 1155 | paper clips |  | 1193 | bird |
| 1167 | horse |  | 1204 | bowl with open lid |
| 1169 | bird |  | 1206 | iron pan |
| 1173 | butterfly 10 |  | 1209 | toaster |
| 1175 | snake |  | 1226 | wodden bowl |
| 1181 | dog 3 |  | 1234 | key |
| 1182 | butterfly |  | 1238 | flat iron |
| 1185 | cat |  | 1239 | scissors |
| 1190 | butterfly 13 moth |  | 1241 | fan |
| 1197 | pot |  | 1244 | pot |
| 1202 | suitcase |  | 1246 | bell |
| 1203 | chinese bowl with spoon |  | 1247 | bunch of keys |
| 1210 | chair |  | 1253 | cooking pot |
| 1212 | bowl with lid |  | 1258 | wall clock |
| 1225 | pocket watch |  | 1261 | plastic container |
| 1245 | flat iron |  | 1272 | glass pot |
| 1251 | paint prush |  | 1275 | pocket watch |
| 1257 | muffin pan |  | 1276 | basket |
| 1259 | cleaning supplies |  | 1292 | leaf 7 |
| 1263 | toaster |  | 1293 | pine cone |
| 1293 | pine cone |  | 1298 | anemone |
| 1296 | holly |  | 1309 | twig |
| 1310 | leaf 13 |  | 1311 | leaf 14 |
| 1312 | leaf 15 |  | 1313 | bicycle |
| 1314 | hot-air ballon |  | 1314 | hot-air ballon |

Images are derived from the Foodpics dataset by Blechert et al., 2014.^6^

**Supplementary Table 2** Comparison of image characteristics between the two image sets, specific to each image category (non-food, and low-calorie and high-calorie foods).

|  | Characteristic | Image Set 1 | Image Set 2 | *p* |
| --- | --- | --- | --- | --- |
| Non-Food | Object Size | 0.29 (0.11) | 0.31 (0.11) | 0.25 |
|  | Brightness | 38.67 (20.13) | 37.32 (16.1) | 0.71 |
|  | Complexity | 0.07 (0.03) | 0.08 (0.03) | 0.21 |
| Low Calorie | Total Protein | 1.69 (1.7) | 1.53 (1.4) | 0.62 |
|  | Total Fat | 0.29 (0.25) | 0.44 (0.69) | 0.16 |
|  | Total Carbs | 4.98 (3.49) | 4.62 (3.28) | 0.59 |
|  | Total Kcal | 30.8 (18.04) | 31.02 (17.99) | 0.95 |
|  | Object Size | 0.32 (0.1) | 0.31 (0.08) | 0.49 |
|  | Brightness | 37.38 (15.12) | 38.19 (15.18) | 0.79 |
|  | Complexity | 0.08 (0.03) | 0.08 (0.03) | 0.86 |
| High Calorie | Total Protein | 25.89 (18.16) | 29.94 (23.22) | 0.33 |
|  | Total Fat | 33.8 (15.52) | 36.6 (23.46) | 0.48 |
|  | Total Carbs | 67.19 (35.59) | 61.52 (44.9) | 0.49 |
|  | Total Kcal | 676.97 (244.51) | 678.52 (245.31) | 0.97 |
|  | Object Size | 0.38 (0.11) | 0.36 (0.08) | 0.5 |
|  | Brightness | 40.82 (15.47) | 38.29 (12.08) | 0.36 |
|  | Complexity | 0.11 (0.04) | 0.1 (0.04) | 0.82 |

**Supplementary Table 3** Significant Clusters for Non-Food (Fasting + Postprandial) contrasts in people living with MND (plwMND)

| Cluster | | Peak | | | | | |
| --- | --- | --- | --- | --- | --- | --- | --- |
| *p* (FWE-corr) | **Size (Voxels)** | **x** | **y** | **z** | **Z** | **T** | **AAL Location** |
| 0 | 17919 | 38 | -56 | -16 | Inf | 17.58 | Fusiform_R |
|  |  | 40 | -75 | -11 | Inf | 17.37 | Occipital_Inf_R |
|  |  | -46 | -70 | -11 | Inf | 16.46 | Occipital_Inf_L |
| 0 | 3290 | -46 | 6 | 30 | 7.07 | 10.81 | Frontal_Inf_Oper_L |
|  |  | -51 | 33 | 15 | 6.63 | 9.59 | Frontal_Inf_Tri_L |
|  |  | -39 | 30 | -14 | 5.97 | 8.02 | Frontal_Inf_Orb_L |
| 0 | 1258 | 42 | 4 | 27 | 6.55 | 9.39 | Frontal_Inf_Oper_R |
|  |  | 35 | 35 | -11 | 5.28 | 6.64 | Frontal_Inf_Orb_R |
|  |  | 50 | 30 | 20 | 5.04 | 6.21 | Frontal_Inf_Tri_R |
| 0 | 748 | -5 | 14 | 54 | 6.52 | 9.31 | Supp_Motor_Area_L |
|  |  | 9 | 6 | 61 | 5.46 | 6.97 | Supp_Motor_Area_R |
|  |  | -8 | -1 | 70 | 5.24 | 6.57 | Supp_Motor_Area_L |
| 0 | 149 | 4 | 4 | 30 | 5.29 | 6.65 | Cingulum_Mid_R |
|  |  | -5 | 11 | 25 | 3.46 | 3.82 | Cingulum_Ant_L |
| 6.00E-03 | 103 | 2 | -56 | -35 | 4.83 | 5.85 | Vermis_9 |
| 0 | 297 | 6 | -34 | 63 | 4.76 | 5.72 | Paracentral_Lobule_R |
|  |  | -8 | -27 | 56 | 4.63 | 5.52 | Paracentral_Lobule_L |
|  |  | 9 | -22 | 56 | 4.23 | 4.89 | Supp_Motor_Area_R |
| 2.40E-02 | 79 | 30 | -70 | -52 | 4.46 | 5.25 | Cerebelum_8_R |
| 0 | 223 | 42 | -32 | 56 | 4.13 | 4.75 | Postcentral_R |
|  |  | 35 | -22 | 56 | 3.92 | 4.44 | Precentral_R |
|  |  | 33 | -22 | 68 | 3.75 | 4.21 | Precentral_R |
| 0 | 8622 | 4 | -75 | 44 | 7.25 | -11.35 | Precuneus_R |
|  |  | -65 | -32 | 25 | 7.07 | -10.8 | SupraMarginal_L |
|  |  | -8 | -77 | 39 | 6.92 | -10.38 | Cuneus_L |
| 0 | 2888 | 57 | -49 | 49 | 6.85 | -10.18 | Parietal_Inf_R |
|  |  | 50 | -49 | 39 | 6.36 | -8.91 | Parietal_Inf_R |
|  |  | 57 | -34 | 37 | 6.11 | -8.34 | SupraMarginal_R |
| 0 | 4098 | 33 | 35 | 44 | 6.53 | -9.33 | Frontal_Mid_R |
|  |  | 6 | 50 | 1 | 6.27 | -8.7 | Frontal_Sup_Medial_R |
|  |  | 6 | 45 | 25 | 5.91 | -7.9 | Cingulum_Ant_R |
| 0 | 378 | -51 | -65 | -42 | 6.02 | -8.13 | Cerebelum_Crus2_L |
|  |  | -44 | -75 | -45 | 4.68 | -5.6 | Cerebelum_Crus2_L |
|  |  | -39 | -49 | -33 | 4.07 | -4.66 | Cerebelum_Crus1_L |
| 0 | 263 | -8 | -58 | -62 | 5.44 | -6.95 | Cerebelum_9_L |
|  |  | 6 | -58 | -62 | 5.2 | -6.49 | Cerebelum_9_R |
|  |  | 6 | -70 | -54 | 3.94 | -4.47 | Cerebelum_8_R |
| 0 | 176 | -32 | 35 | 46 | 4.43 | -5.2 | Frontal_Mid_L |
|  |  | -34 | 45 | 44 | 3.71 | -4.15 | Frontal_Mid_L |
| 1.30E-02 | 89 | 26 | -44 | 70 | 4.15 | -4.78 | Postcentral_R |
|  |  | 30 | -46 | 78 | 3.14 | -3.41 | Postcentral_R |

**Supplementary Table 4** Significant Clusters for Non-Food (Fasting + Postprandial) contrasts in non-neurodegenerative disease (NND) Controls

| Cluster | | Peak | | | | | |
| --- | --- | --- | --- | --- | --- | --- | --- |
| *p* (FWE-corr) | **Size (Voxels)** | **x** | **y** | **z** | **Z** | **T** | **AAL Location** |
| 0 | 15580 | -34 | -68 | -11 | Inf | 19.72 | Fusiform_L |
|  |  | 38 | -94 | 3 | 7.73 | 18.14 | Occipital_Mid_R |
|  |  | 30 | -44 | -16 | 7.48 | 16.45 | Fusiform_R |
| 0 | 2452 | -49 | 30 | 22 | 5.89 | 9.38 | Frontal_Inf_Tri_L |
|  |  | -37 | 30 | -16 | 5.48 | 8.15 | Frontal_Inf_Orb_L |
|  |  | -46 | 4 | 32 | 5.29 | 7.64 | Precentral_L |
| 0 | 691 | 11 | -82 | -35 | 5.65 | 8.63 | Cerebelum_Crus2_R |
|  |  | 28 | -70 | -52 | 5.07 | 7.11 | Cerebelum_8_R |
|  |  | -27 | -70 | -50 | 4.92 | 6.75 | Cerebelum_8_L |
| 0 | 214 | 33 | 33 | -11 | 4.93 | 6.77 | Frontal_Inf_Orb_R |
|  |  | 28 | 28 | 1 | 4.05 | 5.01 | Insula_R |
| 0 | 467 | -8 | 9 | 58 | 4.78 | 6.45 | Supp_Motor_Area_L |
|  |  | -10 | 21 | 51 | 4.7 | 6.26 | Supp_Motor_Area_L |
|  |  | -8 | -3 | 66 | 4.38 | 5.63 | Supp_Motor_Area_L |
| 0 | 781 | 45 | 14 | 30 | 4.62 | 6.1 | Frontal_Inf_Oper_R |
|  |  | 45 | 33 | 15 | 4.28 | 5.43 | Frontal_Inf_Tri_R |
|  |  | 47 | 4 | 30 | 4.23 | 5.35 | Precentral_R |
| 0 | 372 | 9 | -39 | 70 | 4.46 | 5.78 | Paracentral_Lobule_R |
|  |  | 2 | -34 | 70 | 4.4 | 5.65 | Paracentral_Lobule_R |
|  |  | 6 | -27 | 66 | 4.34 | 5.55 | Paracentral_Lobule_R |
| 2.30E-02 | 75 | 6 | 4 | 30 | 4.45 | 5.75 | Cingulum_Mid_R |
|  |  | -5 | 6 | 27 | 3.83 | 4.64 | Cingulum_Ant_L |
| 2.20E-02 | 76 | -22 | -37 | -45 | 4.14 | 5.18 | Cerebelum_10_L |
| 0 | 228 | 35 | -25 | 61 | 4.11 | 5.12 | Precentral_R |
|  |  | 47 | -27 | 49 | 3.91 | 4.77 | Postcentral_R |
|  |  | 33 | -27 | 46 | 3.48 | 4.08 | Postcentral_R |
| 0 | 5909 | -10 | -77 | -4 | 6.45 | -11.36 | Lingual_L |
|  |  | 4 | -56 | 61 | 6 | -9.74 | Precuneus_R |
|  |  | 9 | -77 | -4 | 5.94 | -9.54 | Lingual_R |
| 1.00E-03 | 135 | -29 | -58 | 3 | 5.7 | -8.78 | Precuneus_L |
|  |  | -27 | -46 | -2 | 4.43 | -5.72 | Lingual_L |
| 0 | 1096 | 33 | 26 | 42 | 5.42 | -7.99 | Frontal_Mid_R |
|  |  | 30 | 30 | 51 | 5.19 | -7.4 | Frontal_Mid_R |
|  |  | 33 | 45 | 42 | 5.07 | -7.1 | Frontal_Mid_R |
| 0 | 1105 | 57 | -49 | 51 | 5.07 | -7.1 | Parietal_Inf_R |
|  |  | 57 | -56 | 46 | 4.95 | -6.82 | Parietal_Inf_R |
|  |  | 57 | -49 | 37 | 4.94 | -6.81 | Angular_R |
| 4.00E-03 | 104 | -41 | -80 | 37 | 4.98 | -6.89 | Occipital_Mid_L |
| 0 | 161 | 33 | -53 | 3 | 4.97 | -6.87 | Calcarine_R |
|  |  | 26 | -49 | 8 | 4.2 | -5.28 | Calcarine_R |
|  |  | 18 | -41 | 20 | 3.71 | -4.43 | Cingulum_Post_R |
| 0 | 149 | -63 | -44 | 46 | 4.9 | -6.72 | Parietal_Inf_L |
| 0 | 293 | 4 | -41 | -66 | 4.89 | -6.68 | Cerebelum_9_R |
|  |  | -5 | -61 | -59 | 4.61 | -6.08 | Cerebelum_9_L |
|  |  | -8 | -39 | -64 | 4.33 | -5.53 | Cerebelum_9_L |
| 0 | 323 | 9 | 54 | -2 | 4.67 | -6.21 | Frontal_Med_Orb_R |
|  |  | 9 | 59 | 8 | 3.79 | -4.57 | Frontal_Sup_Medial_R |
|  |  | 4 | 40 | -2 | 3.25 | -3.73 | Cingulum_Ant_R |
| 0 | 142 | -70 | -34 | 20 | 4.5 | -5.85 | Temporal_Sup_L |
|  |  | -56 | -34 | 13 | 3.68 | -4.4 | Temporal_Sup_L |
| 1.00E-03 | 135 | 9 | 40 | 34 | 4.12 | -5.14 | Cingulum_Mid_R |
|  |  | 2 | 45 | 18 | 3.72 | -4.45 | Cingulum_Ant_L |
|  |  | 14 | 47 | 15 | 3.37 | -3.91 | Cingulum_Ant_R |
| 4.00E-03 | 104 | -41 | 38 | 39 | 4.06 | -5.04 | Frontal_Mid_L |
|  |  | -32 | 40 | 44 | 3.94 | -4.82 | Frontal_Mid_L |
| 1.00E-03 | 125 | 66 | -20 | -6 | 4.03 | -4.98 | Temporal_Mid_R |
|  |  | 66 | -18 | -16 | 3.79 | -4.57 | Temporal_Mid_R |
| 8.00E-03 | 91 | -44 | -53 | -42 | 3.76 | -4.52 | Cerebelum_Crus2_L |
|  |  | -49 | -46 | -42 | 3.41 | -3.96 | Cerebelum_Crus2_L |
|  |  | -51 | -65 | -40 | 3.37 | -3.9 | Cerebelum_Crus2_L |

**Supplementary Table 5** Significant Clusters for Food>Non-Food (Fasting + Postprandial) contrasts in people living with MND (plwMND)

| Cluster | | Peak | | | | | |
| --- | --- | --- | --- | --- | --- | --- | --- |
| *p* (FWE-corr) | **Size (Voxels)** | **x** | **y** | **z** | **Z** | **T** | **AAL Location** |
| 0 | 345 | -5 | -32 | 32 | 5.84 | 6.55 | Cingulum_Post_L |
|  |  | -5 | -20 | 32 | 3.84 | 6.29 | Cingulum_Mid_L |
| 0 | 467 | -65 | -22 | 32 | 5.81 | 5.6 | SupraMargil_L |
|  |  | -49 | -39 | 51 | 4.21 | 5.99 | Parietal_Inf_L |
|  |  | -58 | -30 | 44 | 3.57 | 5.87 | Parietal_Inf_L |
| 0 | 273 | -29 | 42 | -18 | 5.8 | 5.51 | Frontal_Inf_Orb_L |
|  |  | -29 | 35 | -9 | 4.74 | 4.12 | Frontal_Inf_Orb_L |
|  |  | -22 | 23 | -21 | 3.49 | 5.58 | Frontal_Inf_Orb_L |
| 0 | 195 | 38 | 6 | -11 | 5.6 | 4.64 | Insula_R |
|  |  | 40 | -3 | 8 | 5.36 | 5.45 | Insula_R |
|  |  | 42 | 14 | -4 | 3.17 | 4.67 | Insula_R |
| 0 | 255 | -39 | -6 | 8 | 5.58 | 5.36 | Insula_L |
|  |  | -39 | 4 | -11 | 4.94 | 4.24 | Insula_L |
|  |  | -51 | -1 | 25 | 3.72 | 5.33 | Precentral_L |
| 0 | 593 | -13 | -101 | -4 | 5.52 | 3.87 | Calcarine_L |
|  |  | -17 | -97 | -11 | 5.42 | 5.27 | Lingual_L |
|  |  | -29 | -77 | -14 | 4.36 | 4.89 | Fusiform_L |
| 0 | 243 | 62 | -18 | 30 | 5.21 | 5.25 | SupraMargil_R |
| 0 | 270 | 18 | -97 | -4 | 5.12 | 5.21 | Calcarine_R |
| 0 | 153 | 26 | 35 | -21 | 4.96 | 4.95 | Frontal_Mid_Orb_R |
|  |  | 23 | 45 | -21 | 4.06 | 4.49 | Frontal_Mid_Orb_R |
| 0 | 412 | 26 | -63 | 42 | 4.72 | 3.91 | Occipital_Sup_R |
|  |  | 18 | -68 | 68 | 4 | 6.55 | Parietal_Sup_R |
|  |  | 26 | -70 | 63 | 3.66 | 6.29 | Parietal_Sup_R |
| 0 | 512 | -20 | -73 | 51 | 4.53 | 5.60 | Parietal_Sup_L |
|  |  | -25 | -65 | 51 | 4.38 | 5.99 | Parietal_Sup_L |
|  |  | -20 | -65 | 42 | 4.33 | 5.87 | Parietal_Sup_L |
| 0 | 169 | 50 | -32 | 46 | 4.18 | 5.51 | SupraMargil_R |
|  |  | 42 | -41 | 56 | 3.72 | 4.12 | Parietal_Inf_R |
| 0 | 610 | 50 | -80 | -2 | 5.24 | -6.57 | Occipital_Mid_R |
|  |  | 52 | -44 | 18 | 4.53 | -5.36 | Temporal_Sup_R |
|  |  | 57 | -61 | 6 | 3.83 | -4.32 | Temporal_Mid_R |
| 0 | 917 | -56 | -65 | 13 | 4.75 | -5.72 | Temporal_Mid_L |
|  |  | -49 | -73 | 22 | 4.65 | -5.55 | Temporal_Mid_L |
|  |  | -65 | -53 | 8 | 4.53 | -5.36 | Temporal_Mid_L |
| 2.90E-02 | 73 | -37 | -37 | -14 | 4.6 | -5.46 | Temporal_Inf_L |
|  |  | -41 | -32 | -18 | 3.88 | -4.4 | Fusiform_L |
| 1.30E-02 | 86 | 11 | -92 | 30 | 4.32 | -5.04 | Cuneus_R |
|  |  | 11 | -97 | 20 | 3.82 | -4.31 | Occipital_Sup_R |
| 7.00E-03 | 97 | 18 | -82 | 1 | 3.94 | -4.47 | Calcarine_R |
|  |  | 18 | -68 | -6 | 3.47 | -3.83 | Lingual_R |

**Supplementary Table 6** Significant Clusters for Food>Non-Food (Fasting + Postprandial) in non-neurodegenerative disease (NND) Controls

| Cluster | | Peak | | | | | |
| --- | --- | --- | --- | --- | --- | --- | --- |
| *p* (FWE-corr) | **Size (Voxels)** | **x** | **y** | **z** | **Z** | **T** | **AAL Location** |
| 0 | 219 | -10 | -101 | -6 | 4.83 | 6.55 | Calcarine_L |
| 2.50E-02 | 67 | 40 | -1 | 3 | 4.71 | 6.29 | Insula_R |
|  |  | 38 | 6 | -11 | 4.37 | 5.60 | Insula_R |
| 1.80E-02 | 71 | 14 | -99 | -4 | 4.57 | 5.99 | Calcarine_R |
| 0.02 | 70 | -39 | 6 | -11 | 4.51 | 5.87 | Insula_L |
|  |  | -39 | -6 | 3 | 4.32 | 5.51 | Insula_L |
|  |  | -37 | -8 | 15 | 3.51 | 4.12 | Insula_L |
| 2.00E-03 | 104 | -32 | -92 | 15 | 4.36 | 5.58 | Occipital_Mid_L |
|  |  | -32 | -82 | 20 | 3.83 | 4.64 | Occipital_Mid_L |
| 0 | 138 | -8 | -46 | 32 | 4.29 | 5.45 | Cingulum_Post_L |
|  |  | -3 | -34 | 34 | 3.85 | 4.67 | Cingulum_Post_L |
| 1.10E-02 | 78 | 28 | -73 | -16 | 4.24 | 5.36 | Cerebelum_6_R |
|  |  | 28 | -87 | -11 | 3.58 | 4.24 | Occipital_Inf_R |
| 8.00E-03 | 83 | 28 | -75 | 46 | 4.22 | 5.33 | Occipital_Sup_R |
|  |  | 23 | -65 | 54 | 3.34 | 3.87 | Parietal_Sup_R |
| 0 | 195 | -22 | -73 | 46 | 4.19 | 5.27 | Parietal_Sup_L |
|  |  | -25 | -75 | 34 | 3.98 | 4.89 | Occipital_Sup_L |
| 3.30E-02 | 63 | -10 | 71 | 1 | 4.18 | 5.25 | Frontal_Sup_Medial_L |
| 3.30E-02 | 63 | -25 | 45 | -18 | 4.16 | 5.21 | Frontal_Mid_Orb_L |
| 1.00E-03 | 119 | -27 | -61 | -16 | 4.01 | 4.95 | Cerebelum_6_L |
|  |  | -32 | -77 | -18 | 3.74 | 4.49 | Cerebelum_Crus1_L |
|  |  | -32 | -87 | -21 | 3.37 | 3.91 | Cerebelum_Crus1_L |
| 0 | 1012 | -56 | -61 | 13 | 4.94 | -6.81 | Temporal_Mid_L |
|  |  | -53 | -51 | 8 | 4.89 | -6.70 | Temporal_Mid_L |
|  |  | -46 | -80 | 3 | 4.86 | -6.63 | Occipital_Mid_L |
| 0 | 861 | 59 | -63 | 13 | 4.77 | -6.41 | Temporal_Mid_R |
|  |  | 50 | -77 | -2 | 4.73 | -6.33 | Occipital_Inf_R |
|  |  | 54 | -68 | 18 | 4.66 | -6.19 | Temporal_Mid_R |
| 0 | 1171 | -3 | -85 | 22 | 4.75 | -6.37 | Cuneus_L |
|  |  | 11 | -87 | 34 | 4.74 | -6.35 | Cuneus_R |
|  |  | 14 | -70 | -4 | 4.63 | -6.12 | Lingual_R |
| 3.30E-02 | 63 | 54 | -6 | -14 | 4.58 | -6.01 | Temporal_Mid_R |
| 9.51E-04 | 115 | -44 | -25 | -23 | 4.43 | -5.72 | Temporal_Inf_L |
|  |  | -41 | -49 | -16 | 3.97 | -4.87 | Fusiform_L |
|  |  | -41 | -32 | -19 | 3.68 | -4.38 | Fusiform_L |
| 1.70E-02 | 72 | -15 | -68 | -4 | 4.20 | -5.29 | Lingual_L |
| 6.34E-03 | 86 | -56 | 11 | -22 | 3.97 | -4.87 | Temporal_Pole_Sup_L |
|  |  | -56 | -1 | -14 | 3.95 | -4.84 | Temporal_Mid_L |

**Supplementary Table 7** Significant Clusters for Food>Non-Food (Fasting) in people living with MND (plwMND)

| Cluster | | Peak | | | | | |
| --- | --- | --- | --- | --- | --- | --- | --- |
| *p* (FWE-corr) | **Size (Voxels)** | **x** | **y** | **z** | **Z** | **T** | **AAL Location** |
| 0 | 355 | 14 | -94 | -9 | 5.18 | 6.46 | Lingual_R |
|  |  | 28 | -75 | -14 | 4.07 | 4.66 | Fusiform_R |
|  |  | 28 | -87 | -14 | 3.93 | 4.46 | Occipital_Inf_R |
| 1.00E-03 | 131 | -39 | -6 | 8 | 5.02 | 6.17 | Insula_L |
| 0.01 | 88 | -65 | -22 | 34 | 4.98 | 6.1 | SupraMargil_L |
| 0 | 147 | -25 | 38 | -16 | 4.72 | 5.67 | Frontal_Mid_Orb_L |
| 0 | 454 | -13 | -101 | -2 | 4.72 | 5.66 | Calcarine_L |
|  |  | -15 | -97 | -11 | 4.58 | 5.43 | Lingual_L |
|  |  | -32 | -61 | -14 | 4.55 | 5.38 | Fusiform_L |
| 5.00E-03 | 100 | 64 | -18 | 32 | 4.69 | 5.61 | SupraMargil_R |
|  |  | 62 | -22 | 44 | 3.27 | 3.58 | SupraMargil_R |
| 3.00E-03 | 106 | 38 | 6 | -11 | 4.51 | 5.33 | Insula_R |
|  |  | 40 | -3 | -2 | 4.5 | 5.31 | Insula_R |
|  |  | 40 | -3 | 10 | 4.37 | 5.11 | Insula_R |
| 0 | 208 | 28 | -63 | 42 | 4.46 | 5.24 | Occipital_Sup_R |
|  |  | 21 | -68 | 68 | 3.85 | 4.34 | Parietal_Sup_R |
|  |  | 30 | -65 | 61 | 3.31 | 3.62 | Parietal_Sup_R |
| 0 | 182 | 52 | -32 | 51 | 4.09 | 4.7 | Parietal_Inf_R |
|  |  | 45 | -32 | 46 | 3.97 | 4.52 | SupraMargil_R |
|  |  | 38 | -30 | 39 | 3.91 | 4.43 | SupraMargil_R |
| 3.00E-03 | 109 | -46 | -37 | 51 | 4.08 | 4.68 | Parietal_Inf_L |
|  |  | -51 | -37 | 44 | 3.8 | 4.28 | Parietal_Inf_L |
|  |  | -49 | -30 | 39 | 3.16 | 3.43 | Parietal_Inf_L |
| 0 | 146 | -22 | -75 | 58 | 3.84 | 4.34 | Parietal_Sup_L |
|  |  | -25 | -65 | 68 | 3.81 | 4.29 | Parietal_Sup_L |
|  |  | -22 | -68 | 49 | 3.75 | 4.21 | Parietal_Sup_L |
| 0 | 748 | -65 | -49 | 3 | 4.86 | -5.89 | Temporal_Mid_L |
|  |  | -51 | -77 | 25 | 4.8 | -5.79 | Angular_L |
|  |  | -53 | -70 | 20 | 4.65 | -5.55 | Temporal_Mid_L |
| 0 | 269 | 11 | -92 | 30 | 4.74 | -5.7 | Cuneus_R |
|  |  | 11 | -94 | 20 | 4.51 | -5.33 | Cuneus_R |
|  |  | 18 | -82 | 3 | 4.27 | -4.95 | Calcarine_R |
| 1.00E-03 | 125 | 47 | -80 | -2 | 4.72 | -5.67 | Occipital_Inf_R |
| 2.00E-03 | 115 | 57 | -68 | 15 | 3.93 | -4.46 | Temporal_Mid_R |
|  |  | 62 | -61 | 10 | 3.84 | -4.34 | Temporal_Mid_R |
|  |  | 52 | -61 | 6 | 3.82 | -4.3 | Temporal_Mid_R |
| 0.03 | 71 | 50 | -51 | 22 | 3.62 | -4.04 | Temporal_Mid_R |
|  |  | 42 | -39 | 18 | 3.57 | -3.97 | Temporal_Sup_R |
|  |  | 54 | -46 | 18 | 3.26 | -3.56 | Temporal_Sup_R |

**Supplementary Table 8** Significant Clusters for Food>Non-Food (Fasting) in non-neurodegenerative disease (NND) Controls

| Cluster | | Peak | | | | | |
| --- | --- | --- | --- | --- | --- | --- | --- |
| *p* (FWE-corr) | **Size (Voxels)** | **x** | **y** | **z** | **Z** | **T** | **AAL Location** |
| 0 | 172 | 11 | 9 | 22 | 4.81 | 6.5 | Caudate_R |
|  |  | -3 | 4 | 30 | 3.99 | 4.91 | Cingulum_Ant_L |
|  |  | 16 | -10 | 25 | 3.79 | 4.56 | Caudate_R |
| 6.00E-03 | 90 | -39 | 6 | -11 | 4.77 | 6.43 | Insula_L |
|  |  | -39 | -6 | 3 | 4.21 | 5.31 | Insula_L |
|  |  | -37 | -8 | 15 | 3.61 | 4.28 | Insula_L |
| 0 | 146 | 35 | 6 | -11 | 4.72 | 6.31 | Insula_R |
|  |  | 35 | -6 | 13 | 4.71 | 6.29 | Insula_R |
|  |  | 40 | -3 | 3 | 3.96 | 4.86 | Insula_R |
| 3.00E-03 | 102 | -25 | -85 | 13 | 4.23 | 5.34 | Occipital_Mid_L |
| 0 | 170 | -8 | -101 | -9 | 4.2 | 5.29 | Calcarine_L |
|  |  | -13 | -101 | -2 | 4.1 | 5.11 | Calcarine_L |
|  |  | -15 | -97 | -11 | 3.95 | 4.84 | Lingual_L |
| 6.00E-03 | 88 | 30 | -73 | -16 | 4.01 | 4.95 | Fusiform_R |
| 4.00E-03 | 94 | -27 | -82 | -14 | 3.8 | 4.58 | Lingual_L |
| 3.00E-03 | 99 | -56 | -63 | 10 | 3.86 | -4.68 | Temporal_Mid_L |
|  |  | -61 | -56 | 10 | 3.8 | -4.59 | Temporal_Mid_L |
|  |  | -51 | -53 | 10 | 3.7 | -4.43 | Temporal_Mid_L |

**Supplementary Table 9** Significant Clusters for High Calorie Food>Non-Food (Fasting) in people living with MND (plwMND)

| Cluster | | Peak | | | | | |
| --- | --- | --- | --- | --- | --- | --- | --- |
| *p* (FWE-corr) | **Size (Voxels)** | **x** | **y** | **z** | **Z** | **T** | **AAL Location** |
| 0 | 689 | -29 | -63 | -14 | 5.25 | 6.58 | Fusiform_L |
|  |  | -15 | -97 | -11 | 5.09 | 6.3 | Lingual_L |
|  |  | -29 | -75 | -14 | 4.94 | 6.03 | Fusiform_L |
| 0 | 578 | 16 | -94 | -11 | 5.04 | 6.21 | Lingual_R |
|  |  | 28 | -51 | -14 | 4.63 | 5.51 | Fusiform_R |
|  |  | 16 | -101 | -2 | 4.46 | 5.25 | Calcarine_R |
| 0 | 149 | -65 | -22 | 34 | 4.99 | 6.11 | SupraMargil_L |
|  |  | -49 | -27 | 34 | 4.24 | 4.91 | SupraMargil_L |
| 3.00E-03 | 113 | -32 | -92 | 20 | 4.88 | 5.93 | Occipital_Mid_L |
| 1.00E-03 | 135 | -25 | 38 | -16 | 4.53 | 5.35 | Frontal_Mid_Orb_L |
|  |  | -29 | 35 | -9 | 4.21 | 4.87 | Frontal_Inf_Orb_L |
| 7.00E-03 | 97 | 35 | -82 | 18 | 4.52 | 5.35 | Occipital_Mid_R |
| 2.20E-02 | 79 | 52 | -58 | -11 | 4.51 | 5.32 | Temporal_Inf_R |
| 6.00E-03 | 102 | 64 | -18 | 32 | 4.46 | 5.25 | SupraMargil_R |
|  |  | 62 | -22 | 44 | 3.8 | 4.27 | SupraMargil_R |
| 1.10E-02 | 90 | -39 | -6 | 6 | 4.28 | 4.98 | Insula_L |
|  |  | -37 | -10 | 13 | 4.11 | 4.72 | Insula_L |
| 2.00E-03 | 124 | -20 | -75 | 56 | 4.27 | 4.95 | Parietal_Sup_L |
|  |  | -20 | -68 | 49 | 3.85 | 4.35 | Parietal_Sup_L |
| 0 | 172 | 38 | -30 | 39 | 4.13 | 4.75 | SupraMargil_R |
|  |  | 42 | -32 | 46 | 4.09 | 4.69 | SupraMargil_R |
|  |  | 50 | -27 | 44 | 3.78 | 4.25 | Postcentral_R |
| 0 | 163 | 26 | -70 | 49 | 4.01 | 4.57 | Parietal_Sup_R |
|  |  | 26 | -70 | 58 | 3.8 | 4.28 | Parietal_Sup_R |
|  |  | 26 | -63 | 39 | 3.7 | 4.15 | Occipital_Sup_R |
| 1.30E-02 | 88 | -46 | -37 | 51 | 3.91 | 4.43 | Parietal_Inf_L |
|  |  | -51 | -37 | 44 | 3.76 | 4.22 | Parietal_Inf_L |
|  |  | -58 | -34 | 51 | 3.66 | 4.09 | Parietal_Inf_L |
| 4.00E-03 | 108 | 50 | -80 | -2 | 4.89 | -5.95 | Occipital_Mid_R |
| 0 | 543 | -65 | -49 | 1 | 4.61 | -5.48 | Temporal_Mid_L |
|  |  | -51 | -80 | 6 | 4.53 | -5.35 | Occipital_Mid_L |
|  |  | -63 | -65 | 15 | 4.31 | -5.01 | Temporal_Mid_L |
| 1.30E-02 | 88 | 11 | -89 | 32 | 4.1 | -4.71 | Cuneus_R |
|  |  | 11 | -94 | 20 | 3.88 | -4.39 | Cuneus_R |
|  |  | 4 | -87 | 25 | 3.16 | -3.43 | Cuneus_L |
| 1.10E-02 | 90 | 59 | -61 | 13 | 3.93 | -4.47 | Temporal_Mid_R |
|  |  | 52 | -63 | 6 | 3.77 | -4.24 | Temporal_Mid_R |
| 6.00E-03 | 102 | 52 | -49 | 20 | 3.59 | -3.99 | Temporal_Mid_R |
|  |  | 62 | -46 | 13 | 3.42 | -3.77 | Temporal_Mid_R |
|  |  | 69 | -44 | 6 | 3.36 | -3.69 | Temporal_Mid_R |

**Supplementary Table 10** Significant Clusters for High Calorie Food>Non-Food (Fasting) in non-neurodegenerative disease (NND) Controls

| Cluster | | Peak | | | | | |
| --- | --- | --- | --- | --- | --- | --- | --- |
| *p* (FWE-corr) | **Size (Voxels)** | **x** | **y** | **z** | **Z** | **T** | **AAL Location** |
| 0 | 393 | 30 | -53 | -11 | 4.67 | 6.21 | Fusiform_R |
|  |  | 30 | -73 | -16 | 4.25 | 5.37 | Fusiform_R |
|  |  | 28 | -85 | -9 | 3.7 | 4.43 | Fusiform_R |
| 8.00E-03 | 87 | -56 | 9 | 34 | 4.62 | 6.11 | Precentral_L |
|  |  | -44 | 6 | 25 | 3.31 | 3.82 | Frontal_Inf_Oper_L |
| 0 | 678 | -29 | -89 | 27 | 4.6 | 6.06 | Occipital_Mid_L |
|  |  | -29 | -89 | 18 | 4.41 | 5.68 | Occipital_Mid_L |
|  |  | -25 | -77 | 37 | 4.25 | 5.37 | Occipital_Mid_L |
| 0 | 261 | -8 | -49 | 32 | 4.56 | 5.99 | Cingulum_Post_L |
|  |  | -8 | -53 | 20 | 4.17 | 5.22 | Precuneus_L |
|  |  | -3 | -34 | 37 | 4.04 | 4.99 | Cingulum_Mid_L |
| 0 | 676 | -8 | -101 | -9 | 4.52 | 5.9 | Calcarine_L |
|  |  | -27 | -77 | -9 | 4.32 | 5.51 | Fusiform_L |
|  |  | -17 | -97 | -11 | 4.23 | 5.34 | Lingual_L |
| 0 | 201 | -8 | 69 | 6 | 4.46 | 5.78 | Frontal_Sup_Medial_L |
|  |  | -10 | 50 | 3 | 4.17 | 5.23 | Cingulum_Ant_L |
|  |  | 2 | 61 | -2 | 3.9 | 4.75 | Frontal_Med_Orb_R |
| 0 | 191 | 35 | -80 | 20 | 4.3 | 5.47 | Occipital_Mid_R |
|  |  | 33 | -89 | 22 | 3.87 | 4.71 | Occipital_Mid_R |
|  |  | 30 | -87 | 37 | 3.26 | 3.75 | Occipital_Mid_R |
| 0 | 203 | 4 | 4 | 30 | 4.1 | 5.09 | Cingulum_Mid_R |
|  |  | -5 | 2 | 30 | 4.02 | 4.95 | Cingulum_Ant_L |
|  |  | 6 | 11 | 25 | 3.79 | 4.57 | Cingulum_Ant_R |
| 5.00E-03 | 92 | 57 | 4 | 30 | 4.07 | 5.04 | Precentral_R |
|  |  | 64 | 2 | 37 | 3.71 | 4.44 | Postcentral_R |
|  |  | 57 | 2 | 49 | 3.68 | 4.38 | Precentral_R |
| 3.40E-02 | 65 | 14 | -97 | -4 | 4.02 | 4.96 | Calcarine_R |
| 3.00E-03 | 102 | -22 | 47 | 37 | 4.02 | 4.96 | Frontal_Sup_L |
|  |  | -20 | 35 | 37 | 3.69 | 4.4 | Frontal_Sup_L |
| 0 | 133 | 30 | -53 | 58 | 3.87 | 4.7 | Parietal_Sup_R |
|  |  | 21 | -68 | 63 | 3.85 | 4.66 | Parietal_Sup_R |
|  |  | 28 | -73 | 58 | 3.4 | 3.95 | Parietal_Sup_R |

**Supplementary Table 11** Significant Clusters for non-neurodegenerative disease (NND) Control > people living with MND (plwMND) Second Level Contrast

| ***p* (FWE-corr)** | **Size (Voxels)** | **x** | **y** | **z** | **Z** | **Location (AAL)** |
| --- | --- | --- | --- | --- | --- | --- |
| **NND Control > MND: Fast > Postprandial: High-Calorie > Non-Food** | | | | | | |
| 0.021 | 75 | 45 | 23 | -26 | 4.27 | Temporal_Pole_Sup_R |
|  |  | 18 | -33 | -33 | 45 | Temporal_Pole_Mid_R |
|  |  | 11 | -28 | -28 | 54 | Temporal_Pole_Mid_R |
| **CNAQ × {MND, NND Control: Fast: Food > Non-Food** | | | | | | |
| 0.001 | 128 | 30 | -61 | -42 | 4.41 | Cerebelum_8_R |
|  |  | -68 | -35 | 4.13 | 38 | Cerebelum_Crus1_R |
|  |  | -65 | -28 | 3.76 | 14 | Cerebelum_6_R |
